# Deacetylation of ACLY Mediates RNA M^6^A‐Modification of NOXA and Promotes Chemoresistance of Colorectal Cancer

**DOI:** 10.1002/advs.202503323

**Published:** 2025-10-02

**Authors:** Jun Wen, Mengqin Shen, Haitao Zhao, Liu Liu, Qian Hua, Xiaoping Zhao, Jianjun Liu, Haizhong Feng, Gang Huang

**Affiliations:** ^1^ Department of Nuclear Medicine, Institute of Clinical Nuclear Medicine, Renji Hospital, School of Medicine Shanghai Jiao Tong University Shanghai 200127 China; ^2^ State Key Laboratory of Systems Medicine for Cancer Renji Hospital Shanghai Cancer Institute Shanghai Jiao Tong University School of Medicine Shanghai 200127 China; ^3^ Shanghai Key Laboratory of Molecular Imaging Shanghai University of Medicine and Health Sciences Shanghai 201318 China; ^4^ Department of Nuclear Medicine The First Affiliated Hospital Zhejiang University School of Medicine Hangzhou 310003 China; ^5^ Department of Nuclear Medicine Shanghai Chest Hospital Shanghai Jiao Tong University School of Medicine Shanghai 200030 China; ^6^ Department of radiology Kunshan Hospital of Traditional Chinese Medicine Kunshan Jiangsu 215300 China

**Keywords:** acetylation, ACLY, chemoresistance, colorectal cancer, m^6^A

## Abstract

Chemoresistance is a major challenge for colorectal cancer (CRC) therapy and is a leading cause of cancer mortality, yet the underlying molecular mechanism remains unclear. ATP citrate lyase (ACLY), a rate‐limiting enzyme of de novo lipid synthesis, plays an important role in tumor progression and chemotherapy. Here, It is demonstrated that deacetylation of ACLY is critical for chemoresistance in CRC. Through proteomic screening acetylated proteins in chemoresistant patient‐derived cells, It is identified that ACLY is deacetylated at K978 site, which induces the relocation of ACLY to the nucleus and promotes its binding to RNA‐binding protein 15 (RBM15). This facilitates N^6^‐methyladenosine (m^6^A) methylation of NOXA (also known as PMAIP1, phorbol‐12‐myristate‐13‐acetate‐induced protein 1) and decreases the stability of NOXA mRNA, resulting in chemoresistance. With the selective inhibitor Santacruzamate A, targeting the deacetylase histone deacetylase 2 (HDAC2) to inhibit the acetylation may enhance the sensitivity of chemoresistance. These findings provide new insights into the mechanism of ACLY deacetylation promoting chemoresistance and suggest a potential therapeutic strategy to mitigate the chemoresistant effects.

## Graphical abstract

This study investigates the deacetylation of ACLY at K978 acts as a novel risk in colorectal cancer chemoresistance. The deacetylation of K978 induces ACLY relocation to the nucleus and promotes its binding to RBM15, a m^6^A methylation modification “writer”. ACLY association with RBM15 facilitates m^6^A methylation NOXA (the pro‐apoptotic gene) and decreases the stability of NOXA mRNA, resulting in chemoresistance.

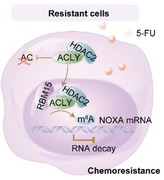

## 1 Introduction

Colorectal cancer (CRC) is the third most common malignant tumor globally, with an increasing incidence in people under 50‐year‐old. It has become the leading cause of cancer death in men and the second leading cause in women.^[^ ^1^ ^]^ Chemotherapy and surgical resection are the main treatments for CRC. Immune checkpoint inhibitors demonstrate notable efficacy in some patients. Recently, a clinical trial showed that 12 CRC patients with mismatch repair (MMR)‐deficiency exhibited high sensitivity to single‐agent programmed cell death protein 1 (PD‐1) blockers and achieved 100% clinical complete response.^[^ ^2^ ^]^ However, only 5% to 10% of patients were MMR‐deficient.^[^ ^3^ ^]^ A large proportion of CRC patients are MMR proficient, and 30% to 60% of these patients are resistant to neoadjuvant chemotherapy (NAC).^[^ ^4^ ^]^ Despite advancements in novel therapeutic approaches, they are not substantially effective in reducing distant recurrence or improving overall survival. The five‐year survival rate for patients with advanced CRC remains below 10%.^[^ ^5^ ^]^ Currently, 5‐fluorouracil (5‐FU) remains one of the most effective and commonly used agents for CRC treatment, and it is the main component of combination chemotherapy regimens. Most patients will receive multiple regimens based on 5‐FU or other fluoropyrimidines.^[^ ^6^ ^]^ Some patients, especially those in advanced stages, are not sensitive to chemotherapeutic agents. Even in tumors that are initially sensitive to chemotherapy, the accumulation of genetic mutations or the activation of certain important signaling pathways by long‐term chemotherapy leads to the development of acquired resistance. The combination of primary and acquired resistance brings about treatment failure in CRC. Hence, pinpointing novel therapeutic targets against CRC advancement and chemoresistance is an urgent requirement.

ACLY expression is upregulated in various malignant tumors, including breast, colorectal, gastric, liver, and prostate cancers.^[^ ^7^, ^8^ ^]^ ACLY is a rate‐limiting enzyme that cleaves citrate to generate acetyl coenzyme A, supporting protein acetylation, in particular that of histone, and de novo lipid synthesis.^[^ ^9^ ^]^ ACLY distributes in the cytoplasm and nucleus, participating in the process of histone acetylation and regulates gene expression.^[^ ^10^ ^]^ In our previous study, ACLY accumulating in the nucleus promotes metastasis of colorectal cancer.^[^ ^11^ ^]^ Moreover, epigenetic modification of ACLY also plays an important role in tumorigenesis. Previous study showed that p53‐binding protein (53BP1) mediates phosphorylation of ACLY, enhances ACLY activity, and further upregulates stem‐loop binding protein (SLBP) promoter acetylation, thereby regulating the DNA double‐strand break (DSB) repair pathway and maintaining genomic integrity.^[^ ^12^ ^]^ Acetylation of ACLY‐lysine (K) 540/546/554 sites competitively inhibits ubiquitination at these lysine sites, promoting lipid synthesis and cell proliferation in tumor cells.^[^ ^13^ ^]^ However, the functions of ACLY epigenetic modifications in cancer chemoresistance are still unknown.

Drug resistance is a major obstacle to tumor therapy, and it is also a crucial cause of poor prognosis. The mechanisms include mutations in therapeutic targets, alterations in drug uptake and efflux, drug inactivation due to molecular structural modifications, epigenetic alterations in DNA, RNA, and proteins, aberrant activation of upstream pathways, and modulation of the disease microenvironment.^[^ ^14^ ^]^ Epigenetics plays an essential role in cancer drug resistance. The advances in DNA methylation, histone modification, chromatin remodeling, and RNA modification have led to a deeper understanding of drug resistance.^[^ ^15^ ^]^ m^6^A methylation is the most common post‐transcriptional modification of eukaryotic mRNAs, accounting for 80% of RNA methylation alterations,^[^ ^16^ ^]^ and it notably influences the processes of mRNA splicing,^[^ ^17^, ^18^ ^]^ transportation,^[^ ^19^ ^]^ and translation.^[^ ^20^ ^]^ m^6^A methylation modification is catalyzed by a “writer” complex consisting mainly of METTL3, METTL14, RBM15, and WTAP. METTL3 is the methyltransferase accountable for conferring m^6^A, while RBM15 and its paralog RBM15B facilitate coupling a large number of mRNAs to the writer complex for methylation.^[^ ^21^ ^]^ Previous studies on the relationship between drug resistance and m^6^A methylation have mainly focused on the differential expression of m^6^A RNA methylation‐modifying enzymes, like METTL3, METTL14, ALKBH5, and there are few studies on the regulation of these molecules.^[^ ^21^, ^22^ ^]^

In this study, we demonstrated that deacetylation of ACLY enhances chemoresistance in CRC. Mechanistically, ACLY‐K978 deacetylation performs a crucial role in enhancing ACLY‐RBM15 protein binding ability and subsequent regulating mRNA m^6^A methylation and stability of NOXA, resulting in the 5‐FU resistance. Collectively, our results uncovered a mechanism governing chemoresistance and indicated that ACLY‐K978 may be a potential therapeutic target to overcome the resistance to 5‐FU for patients with CRC.

## 2 Results

### 2.1 The Important Role of Lysine Deacetylation Modification of ACLY

**Figure 1:**
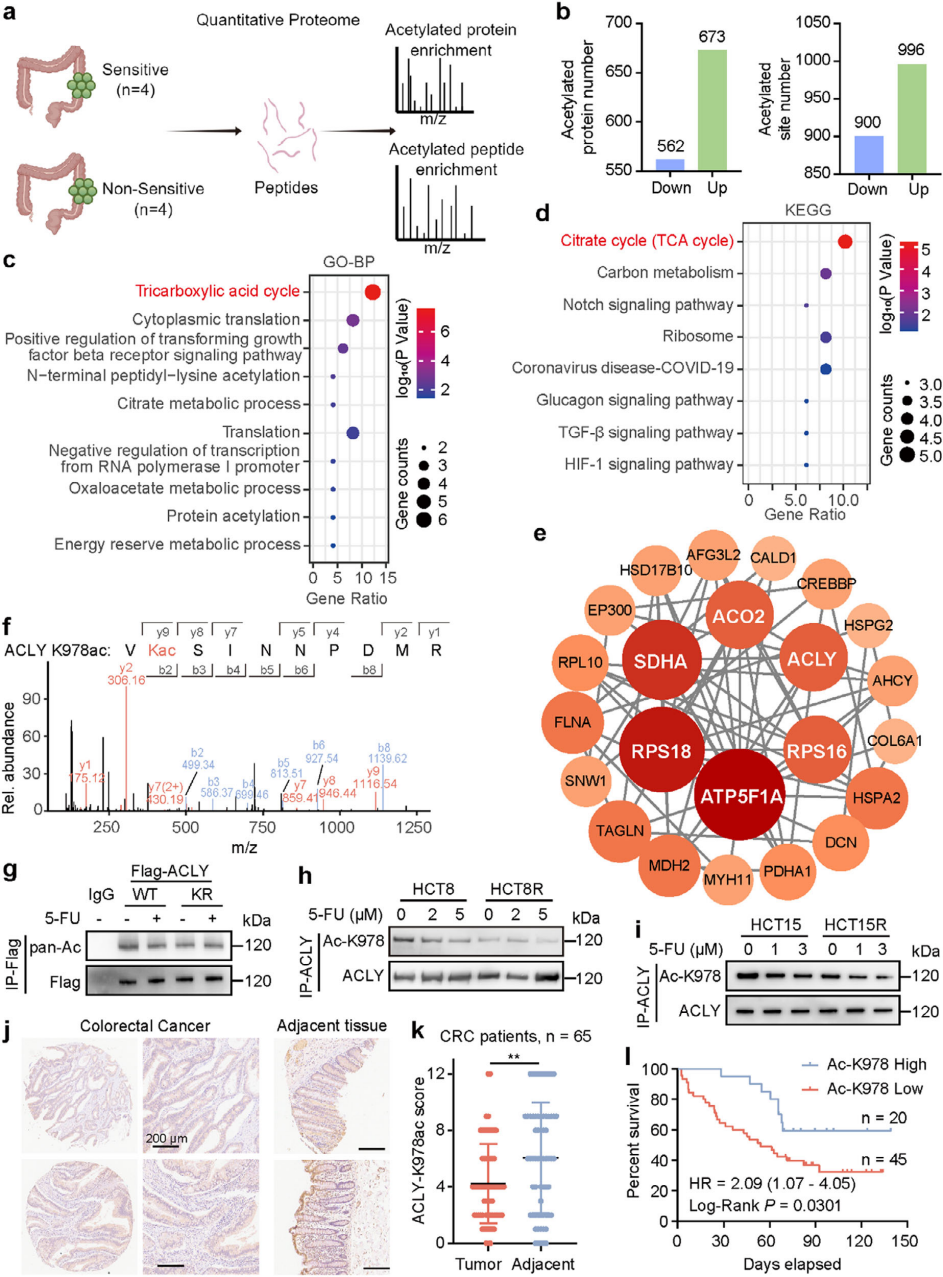
Lysine acetylation profile in cancer chemoresistance and the important role of lysine deacetylation modification of ACLY. a) Schematic representation of quantitative proteomics and acetylation modification analysis of eight postoperative tumor specimens. b) Numbers of differentially expressed acetylated proteins and sites identified by acetylation analysis in sensitive and non‐sensitive groups. c, d) Gene enrichment of biological processes (c) and the Kyoto Encyclopedia of Genes and Genomes (KEGG) pathway enrichment analysis (d) of the proteomics revealed that the Tricarboxylic acid cycle pathway ranked the top significant pathway using a two‐sided hypergeometric test, with *P* values adjusted for multiple comparisons via the Benjamini–Hochberg False Discovery Rate (FDR) method. e) Protein‐protein interaction network of differentially expressed acetylated proteins visualized using Cytoscape software (v3.10.2). f) LC‐MS/MS spectrum of the acetylated ACLY peptide (K978). g) Western blot analysis of K978 acetylation in HCT8 cells transfected with ACLY‐WT/K978R after 5‐FU (1 µM) treatment for 24 h. h, i) Western blot analysis of K978 acetylation in the parental and 5‐FU resistant cells treated with different concentrations of 5‐FU for 24 h. j) Representative immunohistochemical staining for ACLY‐K978ac in tissue microarrays from 65 CRC patients, showing decreased ACLY‐K978ac expression in tumor compared to adjacent normal tissue. k) Comparison of immunohistochemical staining scores of ACLY‐K978ac between tumor and adjacent tissues, ^**^ *p* < 0.01. l) Kaplan‐Meier overall survival analysis using the expression level of ACLY‐K978ac in 65 CRC patients, log‐rank test. Figure 1a was created using Figdraw (www.figdraw.com) and is accompanied by proper copyright authorization.

To investigate the protein acetylation differences associated with intrinsic chemoresistance, we conducted in vitro cultures of postoperative tissue samples from 48 colon cancer patients who had not undergone NAC. Based on variations in drug sensitivity, we selected four chemosensitive and four potentially inherently resistant specimens by culturing tumor tissues in media containing fluorouracil, oxaliplatin, and irinotecan, respectively. We integrated proteomic and acetylation modification analysis on the eight postoperative specimens (**Figure** **1**a). In total, we quantified 1896 lysine acetylation (Kac) sites in 1235 Kac proteins in chemoresistant samples compared to chemosensitive samples (Figure 1b). Then we identified 51 genes with significant acetylation level changes. Gene Ontology (GO) enrichment analysis of biological processes and Kyoto Encyclopedia of Genes and Genomes (KEGG) pathway enrichment analysis revealed that the differentially expressed acetylated genes were enriched in the tricarboxylic acid (TCA) cycle (Figure 1c,d). We performed centrality analysis of the differentially expressed Kac proteins to identify hub genes within the protein‐protein interaction (PPI) network using the STRING database (Figure 1e). ACLY, a key rate‐limiting enzyme in the TCA cycle, was identified as one of the six hub genes. Liquid chromatography‐tandem mass spectrometry (LC‐MS/MS) identified that lysine (K) 978 of ACLY was acetylated (Figure 1f) and the acetylation level was decreased in the potentially inherently resistant specimens (Table S1, Supporting Information). Conservation analysis of ACLY indicated that K978 is a highly conserved site across species from Xenopus tropicalis to Homo sapiens (Figure S1a, Supporting Information). Next, we mutated ACLY K978 to arginine (R) in CRC cells. This K978R mutation notably decreased ACLY acetylation level even in the absence of fluorouracil stimulation (Figure 1g). We then generated K978‐specific antibody (anti‐ACLY‐K978ac) that specifically recognizes ACLY‐K978 acetylation. The specificity of anti‐ACLY‐K978ac was validated using dot blot assays (Figure S1b, Supporting Information). Although the reduction of ACLY‐K978 acetylation was observed after fluorouracil, oxaliplatin, and irinotecan treatment, the trend was most pronounced in the fluorouracil group (Figure S1i, Supporting Information). Given that 5‐FU is a primary component of combination chemotherapy regimens, we established 5‐FU‐resistant CRC cell models (HCT8R and HCT15R) and validated by the Cell Counting Kit‐8 (CCK‐8) assay, Edu assay, and flow cytometric analysis of apoptosis (Figure S1c–h, Supporting Information). Immunoprecipitation (IP) data confirmed a substantial decrease in K978 acetylation levels in chemoresistant cells (Figure 1h,i). To assess the clinical significance of ACLY‐K978 in CRC, we examined its expression levels in CRC patient samples using a tissue microarray on colon or rectal tumor tissues from 65 CRC patients (Table S2, Supporting Information). Statistical analysis shows that the K978 acetylation level of ACLY is significantly lower than that of adjacent tissues (Figure 1j,k). Consistent with the in vitro findings, Kaplan‐Meier analysis indicated that patients with lower acetylation had shorter overall survival in colon cancer (Figure 1l).

### 2.2 ACLY Deacetylation Promotes Chemoresistance

**Figure 2:**
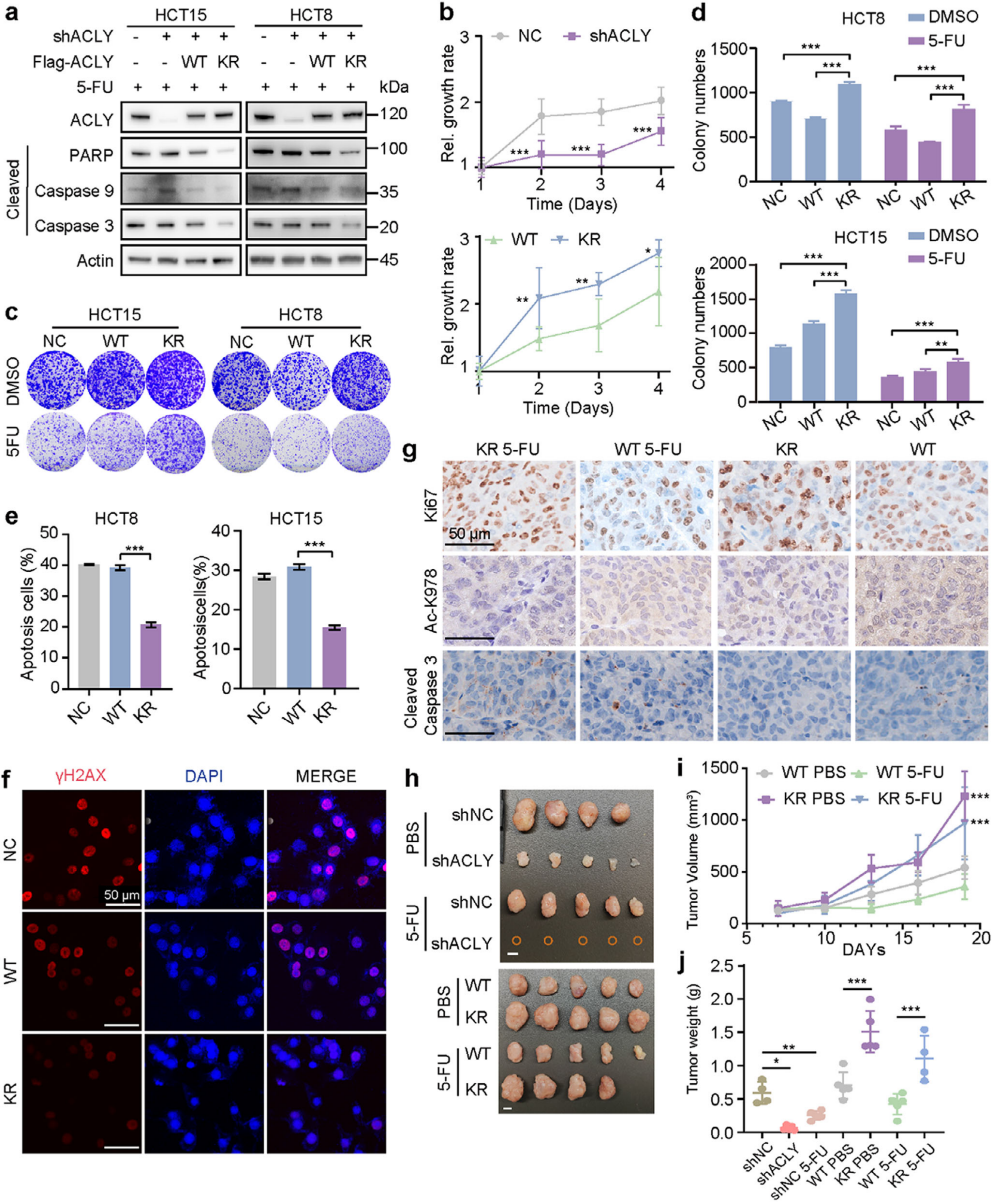
ACLY‐K978 deacetylation promotes chemoresistance. a) Apoptosis‐associated markers were evaluated by western blot in HCT8 and HCT15 cells stably expressing shNC, shACLY, or shACLY‐WT/K978R. b) Cell growth of HCT8 and HCT15 cells stably expressing shNC or shACLY‐WT/K978R (n = 6). c) Representative images of colony formation assays with DMSO or 5‐FU (1 µm) treatment in two CRC cell types. d) Quantification of the colonies (c) (n = 3). e) Apoptotic cells induced by 5‐FU treatment were analyzed by flow Jo (n = 3). f) Immunofluorescence staining of γH2AX (red) and DAPI (blue) in HCT8 shNC/shACLY cells expressing WT/KR (scale bars, 50 µm). g) Representative images of immunohistochemical staining of tumors (scale bars, 50 µm). h) HCT8 tumors were dissected from the BALB/c‐nu mice treated with PBS, or 5‐FU (n = 5/group, scale bars, 5 mm). i) Tumor growth of HCT8 cells in male BALB/c‐nude mice, with PBS or 5‐FU (50 mg kg^−1^) intraperitoneally every three days, beginning on day 7. j) Tumors were dissected and the weight was measured on day 19. Error bars indicate mean ± SD. ^*^ *p* < 0.05, ^**^ *p* < 0.01, and ^***^ *p* < 0.001.

To further explore the role of ACLY‐K978 deacetylation in chemoresistance, we established an ACLY‐knockdown HCT8 cell line and then stably overexpressed ACLY wild‐type (WT)/K978R genes. Western blot analysis confirmed near‐complete knockdown of ACLY, and we observed decreased levels of apoptosis‐related proteins in the KR mutant group (**Figure** **2**a). The CCK‐8 assay revealed that the KR mutant enhanced proliferation ability in these stable cell lines (Figure 2b). Colony formation assay demonstrated KR mutant vitally increased proliferation ability with or without 5‐FU treatment (Figure 2c,d). Flow cytometry analysis showed a significantly reduced proportion of apoptotic cells in the KR mutant group (Figures 2e; S2a, Supporting Information). Consistent with these results, fewer γH2AX foci were observed in KR mutant cells compared to the control following 5‐FU therapy (Figure 2f). These findings suggested that the ACLY‐K978R mutation results in decreased chemotherapy‐induced apoptosis. As expected, genetic knockdown of ACLY or treatment with 5‐FU notably suppressed xenograft tumor growth in immunodeficient BALB/c‐nu mice, as evidenced by a decrease in tumor weight (Figure 2h,i). Tumor burden was remarkably greater in the KR mutant group compared to WT, irrespective of 5‐FU treatment (Figure 2h–j), indicating that ACLY‐K978 drives CRC chemoresistance in a deacetylation‐dependent manner. Histological analysis further confirmed downregulated expression of cleaved caspase‐3 in KR mutant cells subsequent to 5‐FU administration (Figure 2g). A series of functional experiments indicated that the KR mutation promoted 5‐FU resistance of CRC cells both in vitro and in vivo. Collectively, these data demonstrate that K978 is the vital functional Kac site in ACLY that mediates 5‐FU resistance.

### 2.3 Deacetylated ACLY Preferentially Binds to RBM15 Protein

**Figure 3:**
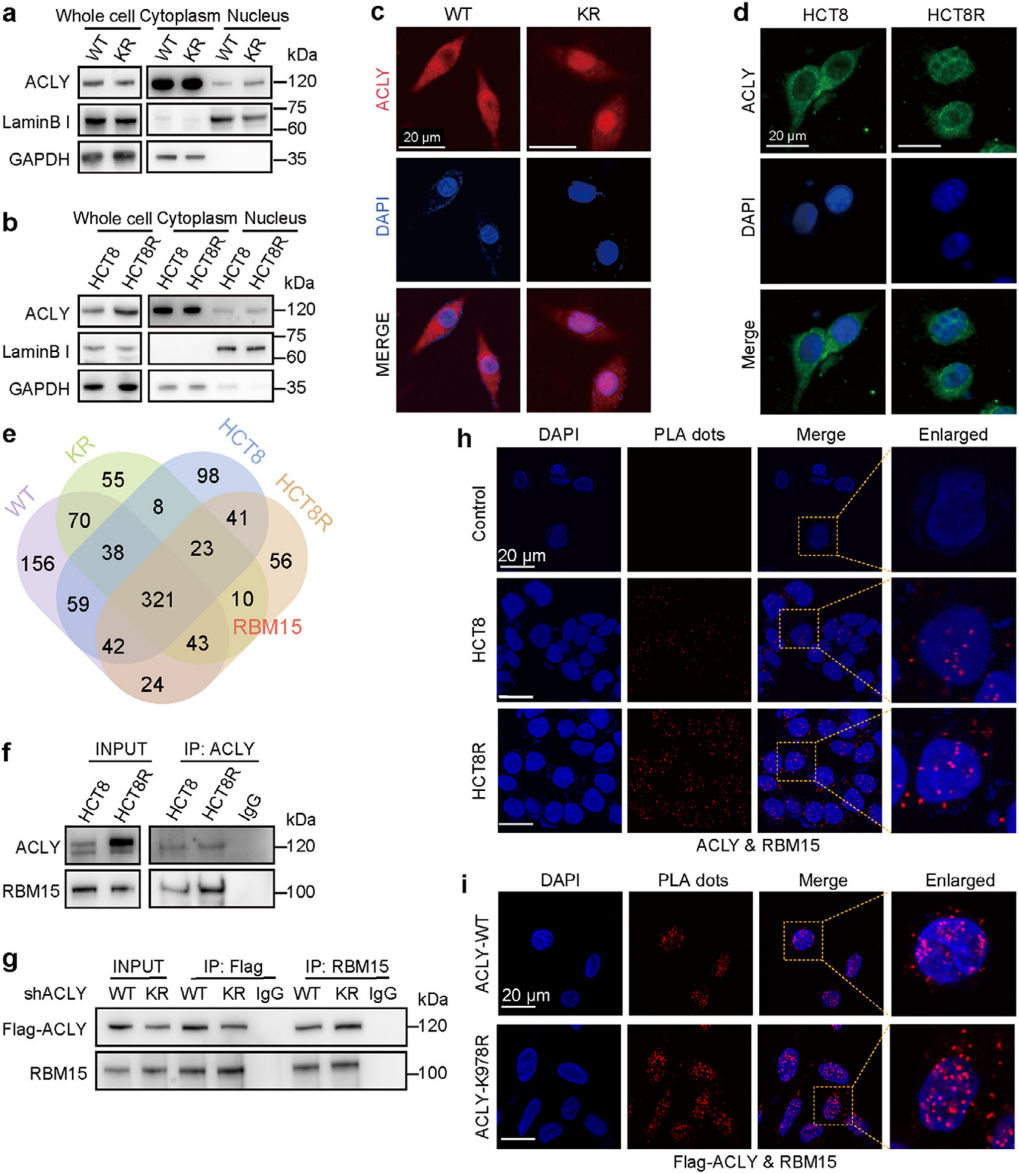
K978‐deacetylated ACLY preferentially binds to RBM15 protein. a) Western blot analysis of ACLY protein localization in WT or KR cells. b) Western blot to detect ACLY protein localization in HCT8 and HCT8R cells. c, d) Immunofluorescence staining showing the subcellular localization of ACLY in CRC cells (scale bars, 20 µm). e) Venn diagram showing the overlap of proteins binding ACLY in different groups (HEK293T cells transfected with Flag‐ACLY‐WT/KR; HCT8 and HCT8R cells transfected with Flag‐ACLY). f) Western blot of input and anti‐ACLY immunoprecipitate (IP) analysis of endogenous ACLY interaction with RBM15 in HCT8 and HCT8R cells. g) Western blot assay to detect interaction between ACLY and RBM15 in HCT8/shACLY cells stably expressing ACLY‐WT/KR. h, i) in situ proximity ligation assay (PLA) demonstrated interaction between ACLY and RBM15 in HCT8 and HCT8R cells (h) or cells stably expressing ACLY‐WT/K978R (i). Positive PLA signals showed ACLY/RBM15 complex, shown as red clusters, with cell nuclei stained blue (scale bars, 20 µm).

Compared to ACLY‐WT, the KR mutant induced the relocation of ACLY to the nucleus (**Figure** **3**a). Immunofluorescence experiments also observed the phenomenon (Figure 3c). Furthermore, ACLY was more likely to accumulate in the nucleus of drug‐resistant HCT8R cells than in HCT8 cells (Figure 3b,d). To explore the mechanism by which KR mutation promotes chemoresistance, we sought to identify differences in proteins that bind to ACLY in the indicated cells using mass spectrometry‐based proteomics. Interestingly, the interaction between ACLY and RBM15 was only detected in KR mutant cells and drug‐resistant HCT8R cells, but not in controls (Figure 3e). The location of ACLY protein‐binding peptides on RBM15 was shown in Figure S2c (Supporting Information). We further investigated the influence of K978 acetylation on the interaction between ACLY and RBM15. We found that K978 deacetylation enhanced the binding of ACLY to endogenous RBM15 (Figure 3g), and a similar phenomenon was observed in HCT8R cells under normal cell culture conditions (Figure 3f). To further characterize the specific ACLY (K978R) ‐RBM15 interaction, we constructed deletion RBM15 mutants (Figure S2d,e, Supporting Information) and co‐expressed Flag‐ACLY and RBM15 truncations in both HCT8 and HCT8R cells. Immunoprecipitation assays demonstrated that ACLY interacted with D1, but not D2‐D4, indicating that ACLY specifically bound to the SPOC domain of RBM15. Notably, the interaction was significantly enhanced in HCT8R cells. Proximity ligation assay (PLA) also showed the enhanced protein‐protein interaction (Figure 3h,i), and relative quantification was shown in Figure S2b (Supporting Information). Collectively, these results indicate that K978 deacetylation is essential for the interaction of ACLY and RBM15.

### 2.4 ACLY Deacetylation Promotes Chemoresistance Depending on RBM15

**Figure 4:**
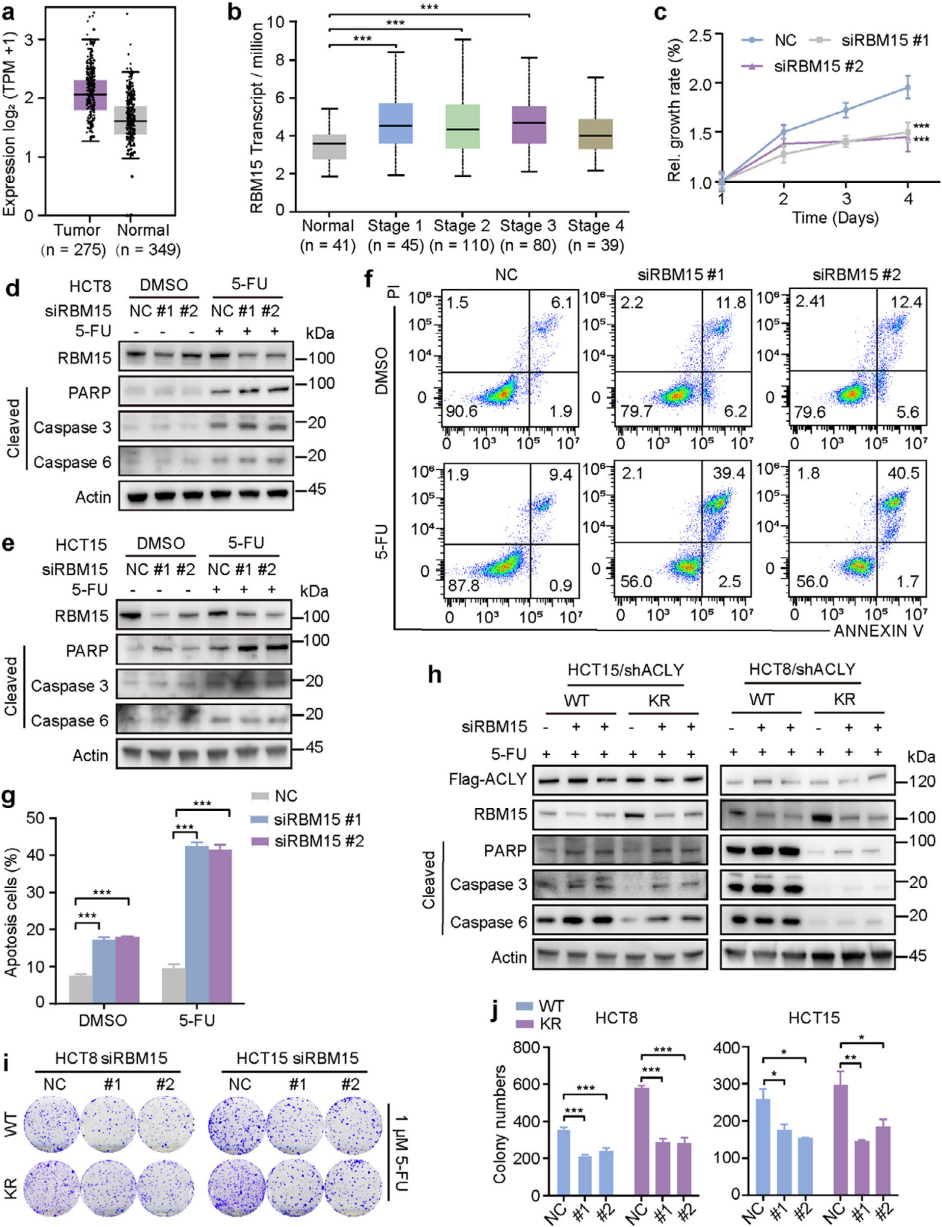
ACLY‐K978 deacetylation promotes chemoresistance depending on RBM15. a) Comparison of RBM15 mRNA expression between normal tissue (n = 349) and CRC (n = 275) from GEPIA2 dataset. b) RBM15 mRNA level in different tumor grades of CRC from UALCAN dataset. c) Cell proliferation assays conducted using HCT8 cells transfected with siNC or siRBM15 #1/2 (n = 6). d, e) Western blot showing the efficiency of RBM15 knockdown and apoptosis‐related protein expression levels after 5‐FU treatment for 24 h. f, g) Apoptotic cells induced by RBM15 knockdown or 5‐FU treatment were analyzed by flow cytometry and flow Jo (n = 3). h) Western blot analysis of apoptosis‐associated proteins in ACLY‐WT/K978R‐expressing HCT8/HCT15 cells with RBM15 knockdown. i) Representative images of colony formation assays in two CRC cell lines transfected with siNC or siRBM15 following 5‐FU treatment. j) Quantification of the colonies in (i) (n = 3). Error bars represent the mean ± SD. For comparisons among multiple groups, one‐way ANOVA was employed. ^*^ *p* < 0.05, ^**^ *p* < 0.01, and ^***^ *p* < 0.001.

RBM15 forms the methyltransferase complex with METTL3, METTL14, and other components.^[^ ^23^ ^]^ RBM15 participates in various biological functions, including proliferation, invasion, migration, and apoptosis, in several cancers, such as glioma,^[^ ^23^ ^]^ lung adenocarcinoma,^[^ ^24^ ^]^ triple‐negative breast cancer,^[^ ^25^ ^]^ gastric cancer,^[^ ^26^ ^]^ prostate cancer,^[^ ^27^ ^]^ myeloid leukemia.^[^ ^28^ ^]^ RBM15 promotes proliferation and migration of colorectal cancer cells.^[^ ^29^ ^]^ To assess the clinical significance of RBM15 in CRC progression, we analyzed RNA sequencing data from the Cancer Genome Atlas (TCGA) using the Gene Expression Profiling Interactive Analysis (GEPIA) platform, which showed upregulation of RBM15 (**Figure** **4**a). RBM15 mRNA levels were significantly increased in stage 1–3 CRC patients compared to controls (Figure 4b). CCK‐8 assay showed that RBM15 knockdown negatively impacted the tumor cell growth (Figure 4c). We knocked down endogenous RBM15 in HCT8 and HCT15 cells using two independent small interfering RNAs (siRNAs) (siRBM15 #1 and #2). The knockdown efficiency of siRBM15 was validated by immunoblotting. Compared to the control group, RBM15 knockdown remarkably increased apoptosis after 5‐FU therapy (Figure 4d,e). Flow cytometry analysis showed a considerably reduced proportion of apoptotic cells in the RBM15 knockdown group (Figure 4f,g). To further explore whether ACLY regulated drug resistance depending on RBM15, we separately knocked down RBM15 in the WT and KR mutant cells and then treated them with 5‐FU. We observed decreased levels of apoptosis‐related proteins in the RBM15 knockdown group, but the KR mutation attenuated apoptosis (Figure 4h). Similarly, the KR mutant increased CRC colony formation after 5‐FU treatment, which depended on RBM15 compared to WT cells (Figure 4i,j).

### 2.5 ACLY‐K978R Participates in the m^6^A Methylation

**Figure 5:**
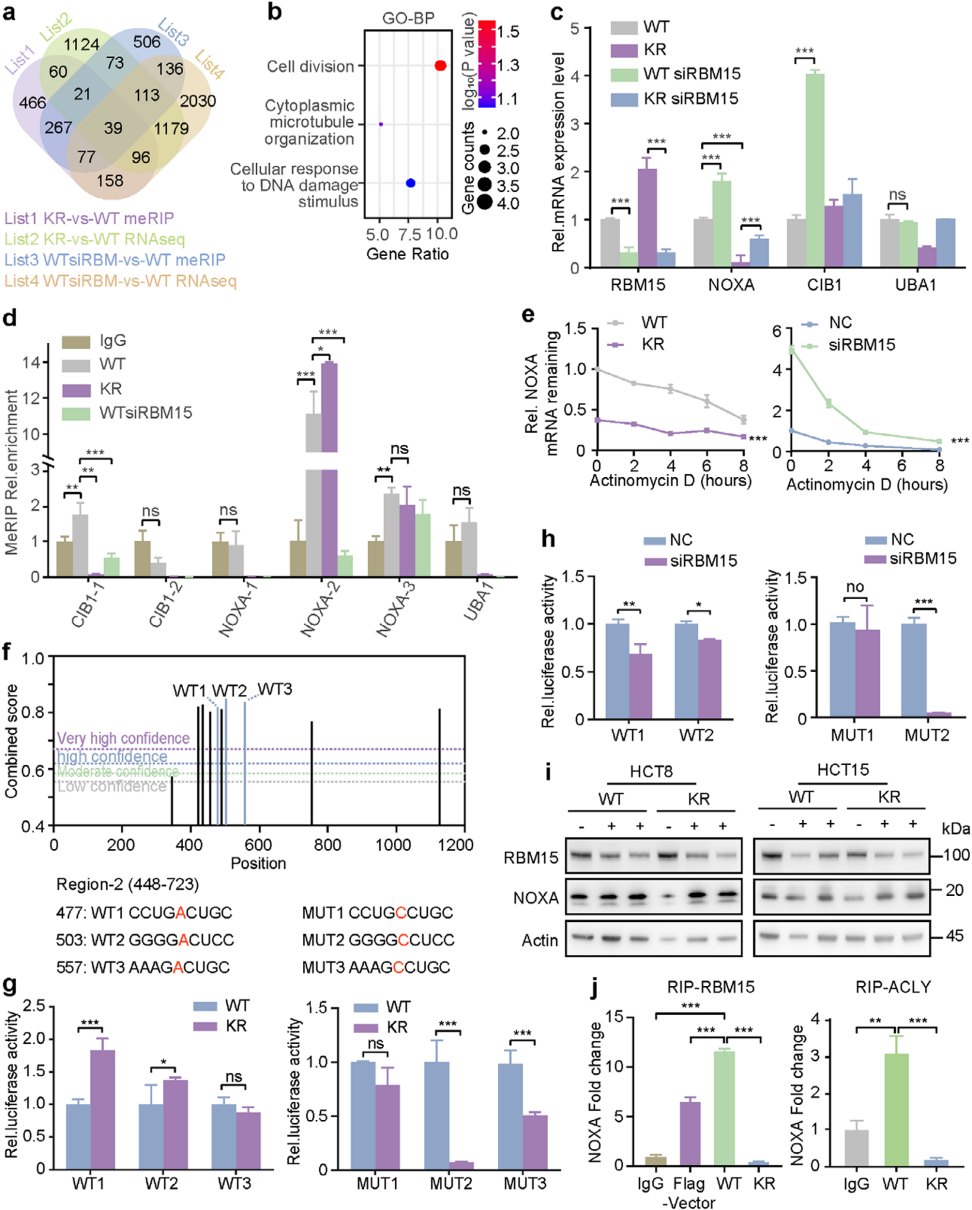
ACLY participates in the m^6^A methylation of NOXA. a) Venn diagram showing the overlap of meRIP‐identified mRNAs and corresponding mRNA levels with significant change in four groups. b) Pathway enrichment analysis of the overlap 39 genes screened from (a). c) Expression levels of mRNAs measured in WT, KR, and relative RBM15 knockdown cells (n = 3). d) MeRIP qRT‐PCR assays measuring m^6^A modification levels in WT, and relative RBM15 knockdown cells, and KR cells (n = 3). e) NOXA mRNA stability analysis in WT/KR or NC/siRBM15 HCT8 cells using actinomycin D treatment (0/2/4/6/8 h) (n = 3). f) Prediction m^6^A modification sites in region‐3 by SRAMP (www.cuilab.cn/sramp) and mutation luciferase reporter plasmids of very high‐confidence prediction sites. g) The luciferase activity in WT/KR cells co‐transfected with wide type/mutated luciferase reporter vectors (WT1/2/3, MUT1/2/3) and si‐NC/siRBM15‐1 (n = 3). h) The luciferase activity in HCT8 cells co‐transfected with wide type/mutated luciferase reporter vectors (WT1/2, MUT1/2) and si‐NC/siRBM15#1 (n = 3). i) Western blot to detect the NOXA protein expression in WT/KR mutation cells with or without RBM15 knockdown. j) RIP assay and subsequent qRT‐PCR analysis of NOXA mRNA were conducted in indicated cells using antibodies against RBM15 or ACLY. Error bars represent the mean ± SD. For comparisons among multiple groups, one‐way ANOVA was employed. ^*^ *p* < 0.05, ^**^ *p* < 0.01, and ^***^ *p* < 0.001; ns, no significance.

There are few reports on whether ACLY can affect mRNA m^6^A levels. To delineate the functional implication of the ACLY‐K978R mutation and identify its downstream targets in CRC chemoresistance, we examined the effect of the KR mutant on mRNA m^6^A levels. Dot blot analysis showed that the KR mutant substantially increased global mRNA m^6^A levels in HCT8 cell lines (Figure S3a, Supporting Information). Next, we performed bulk RNA‐seq and m^6^A‐methylated mRNA immunoprecipitation sequencing (meRIP‐seq), identifying a large number of upregulated individual m^6^A peaks (n = 1395) and a smaller number of downregulated peaks (n = 310) in KR mutant cells compared to WT cells after 5‐FU treatment (Figure S3c, Supporting Information). These findings revealed the role of the KR mutant in promoting overall mRNA m^6^A levels. Bulk RNA‐seq uncovered 2589 upregulated and 135 downregulated genes in KR mutant cells compared to WT cells after 5‐FU therapy (Figure S3e, Supporting Information). Consistent with previous reports,^[^ ^21^, ^30^, ^31^ ^]^ the m^6^A frequency reached its peak near the stop codon (Figure S3b, Supporting Information). Similarly, meRIP‐seq revealed 1132 upregulated and 650 downregulated genes (Figure S3d, Supporting Information), and bulk RNA‐seq uncovered 2688 upregulated and 1182 downregulated genes in RBM15 knockdown cells compared to control cells (Figure S3f, Supporting Information). Thirty‐nine genes were found to be consistently regulated in both KR mutant and RBM15 knockdown cell lines (**Figure** **5**a). Pathway analysis indicated that the shared 39 genes were enriched in a few pathways, such as cell division and cellular response to DNA damage stimulus signaling (Figure 5b). Considering that ACLY‐K978 deacetylation is notably linked to a decreased survival rate of CRC patients (Figure 1l), we focused on the cellular response to DNA damage stimulus pathway genes, including NOXA, CIB1, and UBA1. The m^6^A‐modified transcripts were notably regulated in the four groups as described in Figure S4a–c (Supporting Information).

### 2.6 ACLY‐K978R Mutant Reduces NOXA mRNA Stability Through RBM15‐Dependent Pathway

Among these genes, we selected NOXA (also known as PMAIP1, phorbol‐12‐myristate‐13‐acetate‐induced protein 1) as a candidate target of ACLY‐mediated m^6^A modification for further investigation. The NOXA mRNA level was remarkably upregulated in RBM15 knockdown cells and downregulated in KR mutant cells, but the trend was not observed in CIB1 and UBA1 (Figure 5c). We validated the m^6^A sequence data using meRIP quantitative Real‐time PCR (qRT‐PCR) (Figure 5d). The results showed that the m^6^A‐specific antibody significantly enriched the NOXA mRNA region‐2 compared to the IgG pulldown group, which was more notable in the KR mutant. As expected, knockdown of RBM15 dramatically reduced the m^6^A level of NOXA mRNA. Furthermore, the KR mutant decreased the stability of NOXA mRNA compared to WT cells, whereas the stability of NOXA increased in RBM15 knockdown cells compared to the control group (Figure 5e). Predicted m^6^A sites in region‐2 using SRAMP (www.cuilab.cn/sramp) revealed five very high‐confidence sites. We selected the three highest combined score sites and constructed luciferase reporter plasmids containing mutated sequences of the sites (GAC to GCC) (Figure 5f). WT1 (477A) and WT2 (503A) NOXA‐fused reporter luciferase activity notably increased, but only MUT2 (503C) and MUT3 (557C) NOXA‐fused reporter luciferase activity notably decreased in KR cells (Figure 5g), indicating that the ACLY‐KR mutant regulates NOXA expression through site 2 (503A) modification in region‐2, which was also confirmed in RBM15 knockdown cell lines (Figure 5h). Furthermore, we found that NOXA bound by RBM15 was decreased upon the KR mutant, quantified by RNA immunoprecipitation (RIP) assay and subsequent reverse transcription qPCR, and NOXA bound by ACLY was also decreased upon the KR mutant (Figure 5j). We then tested the effect of the KR mutant on NOXA expression. Consistent with the mRNA level (Figure 5c), the NOXA protein level increased when knocking down RBM15, whereas the impact on NOXA was dramatically weakened by the KR mutant but not WT (Figure 5i). Together, the ACLY‐K978R mutant increases the mRNA m^6^A level and decreases the stability of NOXA.

### 2.7 Inhibiting HDAC2 Deacetylase of ACLY‐K978 Enhances 5‐FU Efficacy

**Figure 6:**
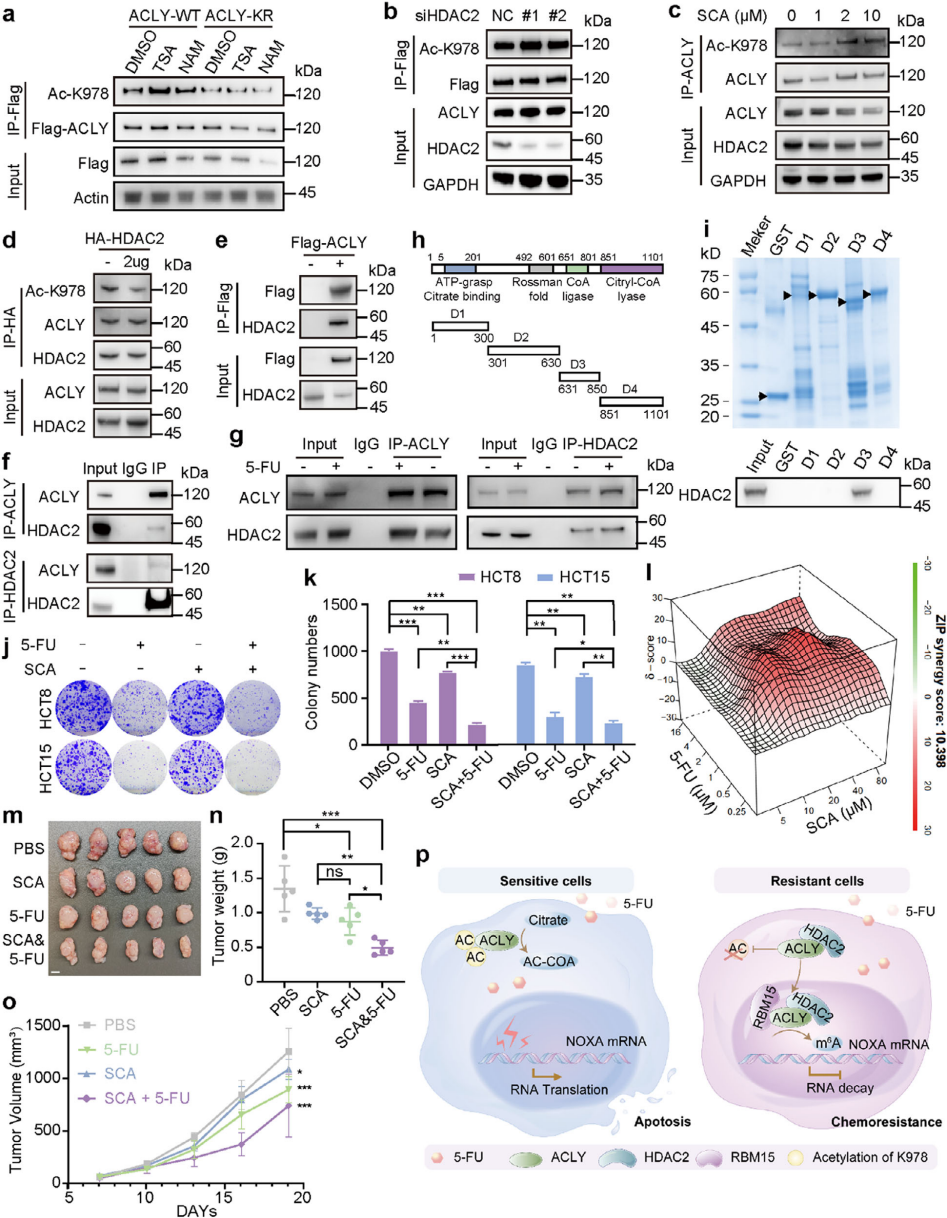
ACLY‐K978 deacetylase HDAC2 enhances the sensitivity of 5‐FU. a) Western blot to detect the ACLY‐K978 acetylation level after treatment with DMSO, NAM, or TSA. b‐d) HCT8 cells were treated with siRNA targeting HDAC2 (b) or Santacruzamate A (SCA) (c) or transfected with HA‐HDAC2 (d), with the acetylation level analyzed by western blot. e, f) IP and western blot to analyze the interaction of ACLY and HDAC2 proteins. g) IP analysis of the interaction between HDCA2 and ACLY proteins in HCT8 cells, with or without 5‐FU treatment (1 µm for 24 h). h) Schematic of GST‐ACLY (full length) and series of deletion mutants was shown. i) GST‐pulldown assay was conducted using recombinant GST‐ACLY deletion mutants (D1‐ D4) and total cell lysates from HCT8. j) Representative images of colony formation assay in HCT8 cells with DMSO, 5‐FU (1 µM), SCA (60 µM), or both treatment (n = 3). k) Quantification of the colonies in (j). l) Calculation and visualization of synergy scores for drug combinations of 5‐FU and SCA by SynergyFinder 2.0 (https://synergyfinder.fimm.fi). m) HCT8 tumors were dissected from the nude mice treated with PBS, 5‐FU (50 mg kg^−1^, every three days), SCA (10 mg kg^−1^, every three days), or both (n = 5/group) intraperitoneally, beginning on day 7. n) Tumors were dissected and the weight was measured on day 19. o) Tumor size was measured every three days during drug treatment. p) The proposed working model was created using Adobe Illustrator 2019. Error bars indicate mean ± SD. For comparisons among multiple groups, one‐way ANOVA was employed. ^*^ *p* < 0.05, ^**^ *p* < 0.01, and ^***^ *p* < 0.001; ns, no significance.

To elucidate the deacetylase responsible for ACLY‐K978, we treated cells with the HDAC inhibitor TSA, which led to an increase in K978 acetylation, whereas the SIRT inhibitor NAM did not (**Figures** **6**a; S5a, Supporting Information). This suggests that the HDAC family is primarily involved in the deacetylation of ACLY‐K978. Subsequently, we selected three selective HDAC inhibitors, including Quisinostat, ACY‐241, and TMP‐195, and observed that only treatment with Quisinostat led to an increase in K978 acetylation (Figure S5b,c, Supporting Information). Thus we targeted K978 deacetylases on HDAC1, HDAC2, HDAC3 and HDAC8 by combining the targets from Quisinostat, ACY‐241 and TMP‐195. Consequently, we detected the protein levels of HDAC1/2/3/8 and found increased protein levels of both HDAC1 and HDAC2 in 5‐FU‐resistant CRC cells (Figure S5d, Supporting Information). Subsequent knockdown experiments demonstrated HDAC2's predominant role in modulating K978 acetylation (Figures 6b; S5e, Supporting Information). Suppression of HDAC2 via knockdown or treatment with the selective inhibitor Santacruzamate A (SCA) enhanced ACLY‐K978 acetylation, while overexpression of HDAC2 reduced it (Figure 6b–d). Using an IP assay, we verified that ACLY interacts with HDAC2 in HCT8 cells transfected with exogenous Flag‐tagged ACLY (Figure 6e), but not HDAC1 (Figure S5f, Supporting Information). Additionally, we confirmed that endogenous ACLY physically associates with HDAC2 (Figure 6f). The interaction between HDAC2 and ACLY was further augmented following 5‐FU treatment (Figure 6g). GST pull‐down assay with GST‐tagged ACLY deletion mutants identified the CoA ligase domain (D3, residues 631–850) of ACLY as the binding region for HDAC2 (Figure 6h,i).

Upregulation of HDAC2 expression has been strongly associated with poor prognosis in colorectal cancer patients.^[^ ^32^, ^33^ ^]^ Notably, HDAC2 inhibitors such as vorinostat (SAHA) and Romidepsin (FK228) have been approved by the FDA for the treatment of cutaneous and peripheral T‐cell lymphoma and are currently under clinical investigation for various solid tumors.^[^ ^32^, ^34^ ^]^ Recent studies have reported that the histone deacetylase inhibitor chidamide, in combination with sintilimab and bevacizumab, significantly improves clinical outcomes in patients with microsatellite‐stable colorectal cancer.^[^ ^35^ ^]^ Consistent with these reports, analysis of TCGA sequencing data via the GEPIA platform confirmed upregulation of HDAC2 in CRC patient tissues (Figure S5g, Supporting Information), with mRNA levels increased across all clinical stages (Figure S5h, Supporting Information). Notably, Kaplan‐Meier analysis of 172 microsatellite stable (MSS) CRC patients revealed that low HDAC2 protein expression was associated with prolonged disease‐free survival (Figure S5i, Supporting Information).

To evaluate the potential clinical application of HDAC2 inhibitors in enhancing fluorouracil sensitivity, we conducted a comprehensive series of functional assays. The colony formation assay demonstrated that the combination of 5‐FU and SCA inhibited tumor growth (Figure 6j,k). Thus we hypothesized that combinatorically targeting of ACLY‐K978 acetylation and 5‐FU could display additional benefit. Indeed, in vitro combinations of inhibitors of HDAC2 and 5‐FU displayed synergistic anti‐CRC efficacy (Figure 6l). Consistent with in vitro findings, tumor burden was reduced by 5‐FU alone, and the effect was more pronounced when combined with SCA (Figure 6m–o).

To investigate the mechanism that HDAC2 inhibition enhanced chemotherapy, we first examined its cytoplasmic expression. To directly address this, we performed subcellular fractionation in both HCT8 and HCT8R cells. Three independent experiments consistently detected HDAC2 expression in cytoplasmic fractions (at lower levels than in nuclear fractions), confirming its presence outside the nucleus (Figure S6a,b, Supporting Information). While the immunofluorescence assay failed to clearly visualize cytoplasmic HDAC2 due to an overwhelming nuclear signal (Figure S6c, Supporting Information). PLA revealed specific ACLY‐HDAC2 interactions in both the cytoplasm and the nuclear. Although interaction signals were predominantly nuclear, discernible cytoplasmic PLA signals were observed – particularly enhanced in 5‐FU‐resistant cells (Figure S6d,e, Supporting Information). These findings support our hypothesis that minor yet functional cytoplasmic HDAC2 pools facilitate ACLY deacetylation prior to nuclear translocation. Subsequently, we tried to evaluate the functional impact of HDAC2 inhibition. Treatment with the HDAC2 inhibitor SCA markedly suppressed proliferation in HCT8R cells, with the inhibitory effect being significantly stronger in the parental HCT8 cells (Figure S7a–c, Supporting Information). The IP assay showed that SCA treatment—both alone and combined with 5‐FU—significantly reduced ACLY‐RBM15 binding, with the combination exhibiting more pronounced effects (Figure S7d,e, Supporting Information). Furthermore, qPCR confirmed markedly elevated NOXA mRNA expression following combination treatment compared to monotherapies (Figure S7f, Supporting Information). These results demonstrate that SCA‐mediated HDAC2 inhibition disrupts the ACLY‐RBM15 complex and enhances NOXA transcription—key downstream events directly regulated by ACLY‐K978 acetylation. Additionally, HDAC2 inhibition altered ACLY localization (Figure S7g, Supporting Information). Treatment with SCA caused a pronounced shift of ACLY from the nucleus to the cytoplasm in HCT8 and HCT8R cells, which was more obvious in HCT8 cells due to its sensitivity to SCA. These data provide the evidence that HDAC2 inhibition can enhance chemotherapy through the ACLY acetylation.

## 3 Discussion

In this study, we identify ACLY‐K978 as a potential therapeutic target for CRC patients. Decrease of ACLY‐K978 acetylation notably reduced the apoptosis induced by chemotherapy in CRC. Mechanistically, the deacetylation induced ACLY accumulation in the nucleus and promoted its binding to RBM15 protein, which facilitated the m^6^A methylation of the target gene NOXA and reduced the stability of NOXA mRNA, resulting in CRC chemoresistance. Furthermore, elevating K978 acetylation via inhibiting HDAC2 resulted in enhanced cellular sensitivity to 5‐FU (Figure 6P).

Epigenetic modifications of ACLY play an important role in tumor progression, including acetylation, and phosphorylation.^[^ ^36^, ^37^, ^38^, ^39^ ^]^ However, the effects of ACLY acetylation in CRC chemoresistance are still unclear. Here we normalize acetylated peptide levels to total protein expression for all CRC samples, and identify that ACLY‐K978 deacetylation enhanced the CRC chemoresistance. The ACLY‐K978R mutation distinctly promoted the proliferation and survival of CRC cells. These findings indicated that ACLY‐K978 is important for the resistance of CRC chemotherapy and may provide a therapeutic target for CRC patients.

To the best our knowledge, we are the first to demonstrate that ACLY regulates m^6^A RNA methylation. Previous study indicated that excessive m^6^A modification led to enhanced ACLY and SCD1 expression in the nonalcoholic fatty liver disease (NAFLD) model.^[^ ^40^ ^]^ Here, we demonstrate that RBM15, a m^6^A methylation modification “writer”, is bound to ACLY. Notably, their interaction was only detected in the K978R mutant or the fluorouracil‐resistant HCT8R cells by LC‐MS/MS ACLY‐K978 deacetylation facilitated the protein interaction of ACLY and RBM15.

NOXA, also known as phorbol‐12‐myristate‐13‐acetate‐induced protein 1 (PMAIP1), plays a crucial role in promoting caspases activation and apoptosis, facilitating mitochondrial outer membrane changes, and the efflux of apoptotic proteins from mitochondria.^[^ ^41^ ^]^ Studies have shown that the up‐ or down‐regulation of NOXA affects the development of malignant tumors, such as colorectal cancer,^[^ ^42^, ^43^ ^]^ melanoma, breast cancer and prostate cancer.^[^ ^44^, ^45^, ^46^ ^]^ NOXA can influence cell apoptosis through both p53‐dependent and p53‐independent pathways. When TP53 is triggered, it directly enhances the transcription of its target gene NOXA, which activates the apoptosis effectors BAX and BAK, resulting in mitochondrial outer membrane permeabilization and the subsequent activation of caspase‐9. This cascade of caspases leads to the breakdown of numerous cellular proteins, facilitating the systematic disassembly of dying cells.^[^ ^41^, ^47^, ^48^ ^]^ NOXA can also regulate cellular apoptosis through p53‐independent pathways. For instance, interferon‐gamma activates ERK, which initiates a stress response involving NOXA to induce apoptosis in melanoma cells.^[^ ^49^ ^]^ In this study, we identified a p53‐independent mechanism that regulates NOXA expression. Specifically, we found that ACLY‐K978 deacetylation facilitates m^6^A methylation of NOXA depending on RBM15, resulting in the downregulation of NOXA mRNA stability. Thus, it reduces sensitivity to chemotherapy in CRC cells.

Chemotherapeutic drugs such as 5‐FU remain the primary treatment of CRC, but drug resistance is the main cause of treatment failure.^[^ ^6^ ^]^ It is essential to comprehend the possible mechanisms behind drug resistance and to identify resistance biomarkers to overcome this challenge and create effective treatments. In this study, we demonstrated ACLY‐K978 plays a critical role in CRC chemoresistance. Then, we discovered the deacetylase of K978 HDAC2. And the specific HDAC2 inhibitor could enhance the sensitivity of 5‐FU in CRC cells.

## 4 Conclusion

In summary, we revealed a new role for ACLY in the progression of CRC and chemoresistance. Deacetylation of ACLY‐K978 promoted chemoresistance in CRC cells. Moreover, ACLY preferentially interacted with RBM15 when K978 deacetylation. And ACLY may upregulate the m^6^A methylation modification of NOXA through RBM15, which in turn reduced the mRNA stability and apoptosis caused by chemotherapy. Inhibiting HDAC2, the deacetylase of ACLY‐K978, is a potential therapeutic strategy for CRC chemoresistance.

## 5 Experimental Section

### Cell Culture

All cell lines were obtained from the Cell Bank of the Chinese Academy of Science (Shanghai, China). HEK293T was cultured in Dulbecco's modified Eagle's medium (DMEM). HCT8, HCT15, and their relative cell lines were cultured in RPMI 1640 medium. The medium was added with 10% fetal bovine serum (Gibco), 100 µg mL^−1^ streptomycin, and 100 units mL^−1^ penicillin. All cells were cultured in a humidified incubator at 37 ℃ with 5% CO_2_.

### Establishment of 5‐Fluorouracil‐Resistant Cell Lines

For colorectal cancer cell lines (HCT8 and HCT15), cells that reached ≈70% confluence in 10 cm dishes were exposed to one‐tenth of the IC_50_ of 5‐FU. The complete culture medium with fresh drug was replaced every three days. Cells were exposed to progressively higher concentrations of 5‐fluorouracil over a period of nearly six months. The IC_50_ value for the cells resistant to 5‐fluorouracil was six times greater than that of the parent cells.

### Transfection and Generating Stable Cell Lines

Transfections were carried out using lipofectamine 2000 (Invitrogen) according to the manufacturer's instruction. Cells were harvested after transfection 48 h and subjected to various assays. Stable knockdown of ACLY was accomplished by using lentivirus‐based short‐hairpin RNA (shRNA). The construction process has been described in the previous study.^[^ ^50^ ^]^ Flag‐ACLY plasmids and the K978 mutation were generated by PCR using the pcDNA 4.0 TO Flag vector. To establish cell lines stably overexpressing, HEK293T cells were transfected with pLV‐CMV‐EGFP‐ACLY (WT/K978R)‐Puro to produce lentivirus, which was harvested 48 h post‐transfection. CRC cells were infected by lentivirus for 6 h and then selected with puromycin (1 µg mL^−1^; MCE) for at least a week to generate stable cell lines. Sequences of siRNA used in this study were listed Table S3 (Supporting Information).

### Xenograft Tumor Model and Treatment

All experiments were performed in accordance with the guidelines of the Animal Ethics Committee of Renji Hospital, School of Medicine, Shanghai Jiao Tong University (approval number: RA‐2021‐233). For tumorigenicity assays, 1 × 10^7^ cells were subcutaneously injected into the right sub‐axillary of 5‐week‐old male BALB/c nude mice (n = 5). When the tumor volume reached 100 mm^3^, the mice were administered with PBS, 5‐FU (50 mg kg^−1^) or SCA (10 mg kg^−1^) every three days via intraperitoneal injection. Tumor size was measured at the indicated time. Tumor volume was calculated according to the formula: 0.5 × length × width^2^. Mice were euthanized when the control group's tumor volume hit 1500 mm^3^, after which the tumor was excised, weighed, and photographed. The tumor tissue was used for immunohistochemistry.

### Clinical Tissue Samples

CRC samples and nearby tissues were collected from the surgical archives at Renji Hospital, School of Medicine, Shanghai Jiao Tong University. The study received approval from the clinical research ethics committee at Renji Hospital, School of Medicine, Shanghai Jiao Tong University (approval number: KY‐2021‐208‐B). Informed written consent was obtained from all participants. None of these patients underwent radiotherapy or chemotherapy before their surgery. Pathological examinations confirmed the diagnosis of all CRC cases.

### Immunohistochemistry

The specimens were preserved using 4% paraformaldehyde (PFA) and subsequently embedded in paraffin. The paraffin‐embedded tissues underwent deparaffinization and rehydration. The samples underwent heat‐induced antigen retrieval, followed by a 10‐min serum blocking at room temperature, and were then incubated with primary antibodies overnight at 4 ℃. The samples were washed the next day and incubated with biotin‐conjugated secondary antibodies for 30 min at room temperature, followed by a DAB peroxidase staining reaction. Nuclear counterstaining was done using hematoxylin. Slides were mounted with a coverslip using a mounting medium.

### Western Blot and IP

Western blot assays were carried out as described in our previous study.^[^ ^11^ ^]^ For IP assays, Anti‐Flag/HA beads (MCE) were incubated with cell extracts overexpressing Flag/HA‐tagged proteins at 4 °C overnight. For the endogenous co‐immunoprecipitation assay, 15 µl protein A/G beads (MCE) were incubated with the indicated antibody and cell extracts in turn. The beads should be washed at least three times with IP buffer after each incubation, in order to completely wash off the remaining antibodies or cell extracts. For acetylation analysis, cells were washed with ice‐cold PBS and then lysed with lysis buffer containing protease and deacetylase Inhibitors. The sample was subjected to a western blot. The antibody specifically recognizing acetylation at lysine‐978 of ACLY was obtained from Shanghai HuiOu Biotechnology Co. Ltd (Shanghai, China). The synthesized peptide IGHRVK (Ac) SINNPD was linked to KLH and used as an antigen to immunize a rabbit. After five immunization doses, anti‐serum was collected. Affinity chromatography was used to eliminate the anti‐non‐K978‐acetylated ACLY antibody from the produced antibody by employing columns with the non‐acetyl‐peptide epitope IGHRVKSINNPD. The anti‐K978‐acetylated ACLY antibody underwent additional purification through affinity chromatography using columns with the matching epitope acetyl‐peptides. The other antibodies used in this study were listed in Table S4 (Supporting Information).

### GST‐Pull Down

Plasmids encoding GST, GST‐ACLY deletion mutants were transformed into BL21 E. coli. The E. coli cells were grown overnight in shaking Luria broth (LB) medium at 37 °C with shaking at 200 rpm. Then fresh LB (200 mL^−1^) was inoculated with pre‐cultured cells in shaking LB medium to reach an OD_600_ of 0.6. Next, isopropyl‐β‐D‐thiogalactoside (IPTG) was added to stimulate protein expression for 12–16 h. Cells were lysed by ultrasound sonification and harvested. Either GST alone or GST‐tagged ACLY fragments were immobilized with glutathione sepharose beads (GE Healthcare) incubated with HA‐HDAC2 overexpressed cell lysates at 4 °C for 3 h. GST beads were washed five times with elution buffer (25 mM glutathione, 50 mM Tris pH 8.8, 200 mM NaCl). Then, the input components and precipitates were combined with SDS loading buffer and heated for 10 min for western blot analysis.

### Nuclear and Cytoplasmic Extraction

The assay was conducted with the NE‐PER nuclear and cytoplasmic extraction Kit (Thermo). Cells were harvested and washed with cold‐PBS, then discarded the supernatant, leaving the cell pellet as dry as possible. To obtain cytoplasmic extract, ice‐cold CER I, II and protease inhibitors were added in order, vortexed, and fully suspended the cell pellet. To obtain nuclear extract, the insoluble fraction containing nuclei was suspended in ice‐cold NER, vortexed, incubated for 40 min, and centrifuged for 10 min. Samples were prepared for western blot analysis.

### Cell Proliferation and Colony Formation Assays

To assess cell viability, cells were seeded in 96‐well plates at a concentration of 1000 cells per well in triplicate and analyzed with the CCK‐8 kit (Yeasen, China) at specified time intervals. In drug sensitivity tests, cells (7000 per well) were placed in 96‐well plates in triplicate and incubated overnight, followed by treatment with designated 5‐FU concentrations. After 48 h, cytotoxicity was evaluated using the CCK‐8 kit. For the colony formation experiment, 2000 cells were placed in a 6‐well plate in triplicate. After around two weeks of growth, surviving colonies were fixed with methanol and stained with 0.1% crystal violet. DMSO or 5‐FU was incorporated into the medium after a week of culturing.

### Edu Assay

The Edu assay was performed with BeyoClick Edu Cell Proliferation Kit with Alexa Fluor 488 (Beyotime, China). Cells were incubated with 5‐ethynyl‐2′ ‐deoxyuridine (Edu) for 2 h in the incubator. Then, cells were fixed using 4% PFA for 15 min, washed three times with 3% BSA in PBS, and then permeabilized with 0.3% Triton X‐100 at room temperature for 15 min. Slides were incubated with click reaction buffer for 30 min at room temperature. For nuclear staining, cells were treated with Hoechst 33342 solution for 10 min. Slides were examined and captured using fluorescence microscopy. The counts of EdU‐positive and Hoechst‐positive cells were used to demonstrate cell proliferation capability.

### Flow Cytometry Analysis of Apoptosis

Apoptosis was evaluated using an Annexin V‐FITC/PI Apoptosis Detection Kit (Yeasen, China). Pancreatic enzymes without EDTA were used to digest cells, which were then collected by centrifuging at 300 rpm for 5 min. Then cells were rinsed twice with cold PBS and then resuspended in 100 µl 1 × binding buffer. Next, they were stained with Annexin V‐FITC and PI staining solution for 10 to 15 min at room temperature in the dark, before being analyzed by flow cytometry.

### Immunofluorescence and Proximity Ligation Assay (PLA)

Cells were seeded onto coverslips of six‐well plates. After 24 h, cells were fixed by 4% PFA, permeabilized with 0.25% Triton X‐100, and blocked in 1% bovine serum albumin (BSA). Then, cells were incubated with the primary antibody at 4 °C overnight, and then incubated with the fluorescent secondary antibody at room temperature for an hour. Slides were sealed using anti‐fluorescence quenching tablets containing 4′,6‐diamidino‐2‐phenylindole (DAPI). and then observed under the Olympus FV3000 confocal microscope to capture images. For the PLA assay, it was carried out using the Sigma‐Aldrich Duolink in situ Red Starter Kit. Briefly, cells were seeded on coverslips, fixed, permeabilized and blocked with BSA. Next, coverslips were incubated with primary and secondary antibodies with PLA probes (PLUS and MINUS) in turn. Subsequently, it was incubated with ligation buffer including ligation enzyme, and polymerase was used to amplify the DNA circle. PLA positive signals are shown in red fluorescence and the nuclei in blue fluorescence. Slides were analyzed by confocal microscope with a 63 × objective.

### Quantitative Real Time‐PCR (qRT‐PCR)

Total RNA was extracted from cells using Trizol reagent (Thermo), followed by cDNA synthesis using Hifair AdvanceFast SuperMix (Yeasen, China). qRT‐PCR assays were performed using SYBR mix (Yeasen, China). The mRNA levels were determined using the ΔCt method and adjusted relative to beta‐actin. The primer sequences employed in this research are provided in Table S2 (Supporting Information).

### m^6^A Dot Blot

For m^6^A dot blot, total RNA extracted from cells was denaturalized in a metal bath at 95 °C for 3 min, then immediately cooled on ice. The mRNA was then transferred onto a membrane in a UV‐light cross‐linking instrument. Membranes were washed in PBST for 5 min with gentle shaking and then incubated with 5% skim milk for an hour at room temperature. After blocking, membranes were incubated with anti‐m^6^A antibody overnight at 4 °C, then incubated with secondary antibodies, and visualization was achieved via enhanced chemiluminescence. The total RNA was detected with methylene blue (MB).

### Mass Spectrometry for Acetylation Modification

Tumor tissues were prepared by trypsin digestion and cell lysates were treated as previously mentioned.^[^ ^39^ ^]^ Proteomic analysis of acetylation was conducted in collaboration with the Jingjie PTM BioLabs, Hangzhou, China. Tryptic peptides were dissolved in solvent A and loaded directly onto a custom‐made reversed‐phase analytical column (25 cm length, 100 µ i.d.). Separation was performed on an EASY‐nLC 1000 UPLC system (Thermo Fisher Scientific) at a constant flow rate of 400 nL min^−1^ using a binary gradient: solvent A (0.1% formic acid, 2% acetonitrile in water) and solvent B (0.1% formic acid, 90% acetonitrile in water). The gradient profile was: 0–24 min^−1^, 7–25% B; 24–32 min^−1^, 25–40% B; 32–37 min^−1^, 40–80% B; 37–40 min^−1^, 80% B.

Eluting peptides were analyzed online using a Q Exactive Plus mass spectrometer (Thermo Fisher Scientific) equipped with a nano‐electrospray ion source (spray voltage: 2000 V). Full MS scans (m/z 350–1800) were acquired in the Orbitrap analyzer at a resolution of 70 000. The top 20 most intense precursor ions were selected for higher‐energy collisional dissociation (HCD) fragmentation (normalized collision energy: 28% or 30%) with a dynamic exclusion window of 15 s. Fragment spectra were acquired in the Orbitrap at a resolution of 17 500. Automatic gain control (AGC) target was set to 5 × 10⁴ for MS/MS, with an intensity threshold of 1.0 × 10⁴ ions s^−1^ and a maximum injection time of 200 ms.

Raw MS/MS data were processed using the MaxQuant software (version 1.5.2.8). Spectra were searched against the human SwissProt database concatenated with a reverse decoy database. Trypsin/P was specified as the cleavage enzyme, allowing up to 4 missed cleavages. Precursor mass tolerance was set to 20 ppm for the initial search and 5 ppm for the main search; fragment ion mass tolerance was 0.02 Da. Carbamidomethyl on Cys was specified as a fixed modification. Variable modifications included protein N‐terminal acetylation, methionine oxidation, and lysine acetylation. TMT‐6plex‐based quantification was performed. Peptide and protein identification false discovery rates (FDR) were controlled at < 1%. To evaluate acetylation levels, lysine acetylation was normalized against the abundance of the corresponding total protein. This normalized acetylation level served as the primary measure.

### Proteomic Analysis

To detect ACLY‐interacting proteins, HCT8/shACLY cells stably expressing Flag‐ACLY or Flag‐ACLY‐K978R were subjected to IP assays with anti‐Flag magnetic beads. HCT8 and HCT8R cells transfected with Flag‐ACLY were also subjected to IP assays with anti‐Flag magnetic beads. Proteins that were immunoprecipitated were separated using SDS–polyacrylamide gel electrophoresis and then visualized with Coomassie blue staining, and then analyzed by LC‐MS/MS at Orbitrap Exploris 480 (Thermo). All sequence data were deposited into open datasets. The mass spectrometry proteomics data have been deposited to the ProteomeXchange Consortium (https://proteomecentral.proteomexchange.org) via the iProX partner repository the dataset identifier PXD059071.

### MeRIP qRT‐PCR and Sequencing

The Magna MeRIP m^6^A Kit (Merck) was utilized for identification and transcriptome‐wide profiling of N^6^‐methyladenosine (m^6^A) RNA methylation sites. Briefly, total RNA was isolated and fragmented into small pieces (≈100 nucleotides). These RNA fragments were then subjected to immunoprecipitation using the m^6^A antibody (Synaptic Systems) bound to magnetic beads. After thorough washing to remove unbound RNA, the immunoprecipitated RNA was eluted and purified. The purified RNA was either analyzed by qRT‐PCR or used for meRIP sequencing to map the m^6^A methylation sites across the transcriptome. A qualified library for sequencing was obtained and sequenced using Illumina NovaSeq 6000 by Gene Denovo Biotechnology Co. (China). All meRIP sequencing data generated in this study have been deposited in the Genome Sequence Archive (GSA‐human) in the National Genomics Data Center (NGDC), China National Center for Bioinformation, under accession number [HRA009901]. All other datasets used in the study are publicly available. The primers for the MeRIP qPCR assay were listed in Table S2 (Supporting Information).

### mRNA Stability

To measure mRNA stability, cells were treated with 2 µg mL^−1^ Actinomycin D (Selleck) for 0, 2, 4, 6, or 8 h. Cells were collected and extracted RNA. The same volume of RNA was reverse transcribed. Quantitative RT‐PCR was performed to measure the target mRNA levels.

### Luciferase Reporter Assay

For miRNA target validation, reporter plasmids containing pGL3‐NOXA‐wild‐type (WT) and pGL3‐NOXA‐mutant sequence (the putative m^6^A site was substituted with C) were synthesized by Tsingke Biological Technology (Beijing, China). Firefly and renilla luciferase plasmid were transfected into cells at a ratio of 30:1 using lipofectamine 2000. After 24 h, firefly and renilla luciferase activities were analyzed using the Dual‐Luciferase Reporter Assay System (Promega).

### RNA Immunoprecipitation (RIP) qPCR

RIP assay was performed with the Magna RIP Kit (Millipore). Cells were rinsed with cold PBS, and lysed in RIP Lysis Buffer supplemented with protease and RNase inhibitors. The lysate was then processed by thawing, centrifuging, and transferring the supernatant to a fresh tube. Protein A/G beads were resuspended, washed, and incubated with the specific antibody (e.g., Anti‐RBM15) and rabbit IgG as a control. Subsequently, the cell lysate was mixed with the bead‐antibody complex and incubated overnight at 4 ℃ to allow for the binding of RNA‐protein complexes to the beads. Following incubation, the beads were washed to remove unbound materials. RNA elution was performed by incubating the beads with RIP Wash Buffer, SDS, and Proteinase K, which digested the proteins and released the bound RNA into the supernatant. The RNA was then purified by adding phenol‐chloroform‐isoamyl alcohol, vortexing, centrifuging, and collecting the aqueous phase. After precipitation with ethanol and RNase‐free salts, the RNA was washed, air‐dried, and dissolved in RNase‐free water. The concentration of the purified RNA was determined using a NanoDrop spectrophotometer, and the RNA samples were analyzed by qRT‐PCR. All steps were carried out in a nuclease‐free environment using RNase‐free reagents to ensure the integrity and purity of the RNA.

### Statistical Analysis

All statistical analysis were conducted with GraphPad Prism 8. Results are shown as means ± SD from at least three independent experiments. To compare the means of more than two groups, a one‐way ANOVA was applied. The mean values of the two groups were compared using Student's t test. For meRIP analyses, differentially methylated mRNAs were prioritized using unadjusted *P* values and log_2_ fold change (log_2_FC) thresholds. The Kaplan‐Meier plot was used to draw the survival curve, and comparisons were made with the log‐rank test. ^*^ *p* < 0.05, ^**^ *p* < 0.01, and ^***^ *p* < 0.001; ns, no significance.

## Conflict of Interest

The authors declare no competing Interest.

## Author Contributions

Conceptualization was done by J.W.; methodology by J.W. and M.S.; validation by J.W., M.S., and H.Z.; formal analysis by J.W., L.L., and Q.H.; investigation by J.W., J.L., X.Z., H.F., and G.H.; writing of the original draft by J.W.; writing—review and editing by J.W., J.L., X.Z., H.F., and G.H.; visualization by J.W., L.L., and Q.H.; funding acquisition by J.W., M.S., H.Z., L.L., Q.H., H.F., and G.H.; resources provided by H.F. and G.H.; and supervision by J.L., H.F., and G.H.

## Supporting information



Supporting Information

## Data Availability

The data that support the findings of this study are available from the corresponding author upon reasonable request. The data are not publicly available due to privacy or ethical restrictions.

## References

[1] R. L. Siegel , A. N. Giaquinto , A. Jemal , Ca‐Cancer J. Clin. 2024, 74, 12. 38230766 10.3322/caac.21820

[2] A. Cercek , M. Lumish , J. Sinopoli , J. Weiss , J. Shia , M. Lamendola‐Essel , I. H. El Dika , N. Segal , M. Shcherba , R. Sugarman , Z. Stadler , R. Yaeger , J. J. Smith , B. Rousseau , G. Argiles , M. Patel , A. Desai , L. B. Saltz , M. Widmar , K. Iyer , J. Zhang , N. Gianino , C. Crane , P. B. Romesser , E. P. Pappou , P. Paty , J. Garcia‐Aguilar , M. Gonen , M. Gollub , M. R. Weiser , et al., N Engl J Med. 2022, 386, 2363. 35660797 10.1056/NEJMoa2201445 PMC9492301

[3] O. I. Alatise , G. C. Knapp , A. Sharma , W. K. Chatila , O. A. Arowolo , O. Olasehinde , O. C. Famurewa , A. D. Omisore , A. O. Komolafe , O. O. Olaofe , A. I. Katung , D. E. Ibikunle , A. A. Egberongbe , S. A. Olatoke , S. O. Agodirin , O. A. Adesiyun , A. Adeyeye , O. A. Kolawole , A. O. Olakanmi , K. Arora , J. Constable , R. Shah , A. Basunia , B. Sylvester , C. Wu , M. R. Weiser , K. Seier , M. Gonen , Z. K. Stadler , Y. Kemel , et al., Nat. Commun. 2021, 12, 6821. 34819518 10.1038/s41467-021-27106-w PMC8613248

[4] Y. Deng , P. Chi , P. Lan , L. Wang , W. Chen , L. Cui , D. Chen , J. Cao , H. Wei , X. Peng , Z. Huang , G. Cai , R. Zhao , Z. Huang , L. Xu , H. Zhou , Y. Wei , H. Zhang , J. Zheng , Y. Huang , Z. Zhou , Y. Cai , L. Kang , M. Huang , X. Wu , J. Peng , D. Ren , J. Wang , J. Clin. Oncol. 2019, 37, 3223. 31557064 10.1200/JCO.18.02309 PMC6881102

[5] C. Du , D. Huang , Y. Peng , Y. Yao , Y. Zhao , Y. Yang , H. Wang , L. Cao , W.‐G. Zhu , J. Gu , Cancer Lett. 2017, 400, 183. 28465257 10.1016/j.canlet.2017.04.033

[6] S. Vodenkova , T. Buchler , K. Cervena , V. Veskrnova , P. Vodicka , V. Vymetalkova , Pharmacol. Ther. 2020, 206, 107447. 31756363 10.1016/j.pharmthera.2019.107447

[7] W. Xiang , H. Lv , F. Xing , X. Sun , Y. Ma , L.u Wu , G. Lv , Q. Zong , L. Wang , Z. Wu , Q. Feng , W. Yang , H. Wang , Sci. Adv. 2023, 9, adi2465. 10.1126/sciadv.adi2465 PMC10699784 38055816

[8] Y. Chen , J. Shen , M. Yuan , H. Li , Y. Li , S. Zheng , B.o Han , C. Zhang , S. Liu , Q. Sun , J. Wu , J. Adv. Res. 2025, 67, 331. 38295877 10.1016/j.jare.2024.01.028 PMC11725148

[9] K. H. G. Verschueren , C. Blanchet , J. Felix , A. Dansercoer , D. De Vos , Y. Bloch , J. Van Beeumen , D. Svergun , I. Gutsche , S. N. Savvides , K. Verstraete , Nature 2019, 568, 571. 30944476 10.1038/s41586-019-1095-5

[10] K. E. Wellen , G. Hatzivassiliou , U. M. Sachdeva , T. V. Bui , J. R. Cross , C. B. Thompson , Science 2009, 324, 1076. 19461003 10.1126/science.1164097 PMC2746744

[11] J. Wen , X. Min , M. Shen , Q. Hua , Y. Han , L.i Zhao , L. Liu , G. Huang , J. Liu , X. Zhao , J. Exp. Clin. Cancer Res. 2019, 38, 401. 31511060 10.1186/s13046-019-1391-9 PMC6740040

[12] T. Wu , S. Jun , E.‐J. Choi , J. Sun , E.‐B. Yang , H.‐S. Lee , S.‐Y. Kim , N. A. Fahmi , Q. Jiang , W. Zhang , J. Yong , J.‐H. Lee , H. J. You , Nucleic Acids Res. 2022, 50, 1465. 35037047 10.1093/nar/gkab1300 PMC8860602

[13] R. Lin , R. Tao , X. Gao , T. Li , X. Zhou , K.‐L. Guan , Y. Xiong , Q.‐Y. Lei , Mol. Cell 2013, 51, 506. 23932781 10.1016/j.molcel.2013.07.002 PMC4180208

[14] X. Sun , Y. Zhang , H. Li , Y. Zhou , S. Shi , Z. Chen , X. He , H. Zhang , F. Li , J. Yin , M. Mou , Y. Wang , Y. Qiu , F. Zhu , Nucleic Acids Res. 2023, 51, D1263. 36243960 10.1093/nar/gkac812 PMC9825618

[15] H. Zhuang , B. Yu , D. Tao , X. Xu , Y. Xu , J. Wang , Y. Jiao , L. Wang , Molecular Cancer 2023, 22, 91. 37264402 10.1186/s12943-023-01782-2 PMC10233906

[16] P. Boccaletto , F. Stefaniak , A. Ray , A. Cappannini , S. Mukherjee , E. Purta , M. Kurkowska , N. Shirvanizadeh , E. Destefanis , P. Groza , G. Avsar , A. Romitelli , P. Pir , E. Dassi , S. G. Conticello , F. Aguilo , J. M. Bujnicki , Nucleic Acids Res. 2022, 50, D231. 34893873 10.1093/nar/gkab1083 PMC8728126

[17] Y. Wu , M. Jin , M. Fernandez , K. L. Hart , A. Liao , X. Ge , S. M. Fernandes , T. McDonald , Z. Chen , D. Röth , L. Y. Ghoda , G. Marcucci , M. Kalkum , R. K. Pillai , A. V. Danilov , J. J. Li , J. Chen , J. R. Brown , S. T. Rosen , T. Siddiqi , L. Wang , Blood Cancer Discovery 2023, 4, 228. 37067905 10.1158/2643-3230.BCD-22-0156 PMC10150290

[18] A. Baek , G.‐E. Lee , S. Golconda , A. Rayhan , A. A. Manganaris , S. Chen , N. Tirumuru , H. Yu , S. Kim , C. Kimmel , O. Zablocki , M. B. Sullivan , B. Addepalli , L. Wu , S. Kim , Nat. Microbiol. 2024, 9, 1340. 38605174 10.1038/s41564-024-01638-5 PMC11087264

[19] S.‐K. Shan , X. Lin , F. Wu , C.‐C. Li , B. Guo , F.‐X.‐Z. Li , M.‐H. Zheng , Y. Wang , Q.‐S. Xu , L.‐M. Lei , K.‐X. Tang , Y.‐Y. Wu , J.‐Y. Duan , Y.‐C. Cao , Y.‐L. Wu , C.‐M. Tan , Z.‐H. Liu , Z.‐A. Zhou , X.‐B. Liao , F. Xu , L.‐Q. Yuan , Bioact. Mater. 2024, 42, 52. 39280584 10.1016/j.bioactmat.2024.08.021 PMC11399808

[20] T. Berulava , E. Buchholz , V. Elerdashvili , T. Pena , M.d R. Islam , D. Lbik , B. A. Mohamed , A. Renner , D. von Lewinski , M. Sacherer , K. E. Bohnsack , M. T. Bohnsack , G. Jain , V. Capece , N. Cleve , S. Burkhardt , G. Hasenfuss , A. Fischer , K. Toischer , Eur. J. Heart Failure 2020, 22, 54. 10.1002/ejhf.1672 31849158

[21] J. Zhang , J. Wei , R. Sun , H. Sheng , K. Yin , Y. Pan , R. Jimenez , S. Chen , X.‐L. Cui , Z. Zou , Z. Yue , M. J. Emch , J. R. Hawse , L. Wang , H. H. He , S. Xia , B. Han , C. He , H. Huang , Mol. Cell 2023, 83, 2692. 37478845 10.1016/j.molcel.2023.06.024 PMC10427207

[22] S. Pan , Y. Deng , J. Fu , Y. Zhang , Z. Zhang , X. Qin , Int. J. Oncol. 2022, 60, 14. 35014676 10.3892/ijo.2022.5304 PMC8759347

[23] X. Guo , W. Qiu , C. Wang , Y. Qi , B. Li , S. Wang , R. Zhao , B.o Cheng , X. Han , H. Du , Z. Gao , Z. Pan , S. Zhao , G. Li , H. Xue , Cancer Res. 2024, 84, 372. 37963207 10.1158/0008-5472.CAN-23-0609

[24] H. Li , Y. Li , X. Zheng , F. Chen , S. Zhang , S. Xu , Y. Mu , W. Shen , J. Tong , H. Chen , Z. Hu , J. Zhang , K. Qiu , W. Chen , X. Cheng , G. Xu , Oncogene 2024, 44, 307. 39528815 10.1038/s41388-024-03220-z PMC11779629

[25] S. H. Park , J.‐S. Ju , H. Woo , H. J. Yun , S. B. Lee , S.‐H. Kim , B. Gyorffy , E.‐J. Kim , H. Kim , H. D. Han , S.‐I. Eyun , J.‐H. Lee , Y.‐Y. Park , Exp. Mol. Med. 2024, 56, 1373. 38825643 10.1038/s12276-024-01235-w PMC11263342

[26] X. Cai , X. Li , M. Zhang , Z. Dong , Y. Weng , W. Yu , Biochim Biophys Acta Mol Cell Biol Lipids. 2024, 1870, 159580. 39549859 10.1016/j.bbalip.2024.159580

[27] Y. Ying , Y. Wu , F. Zhang , Y. Tang , J. Yi , X. Ma , J. Li , D. Chen , X. Wang , X. Liu , B. Liu , J. Luo , X. Zheng , L. Xie , Molecular Cancer 2024, 23, 79. 38658974 10.1186/s12943-024-01994-0 PMC11041046

[28] X. Dou , Y.u Xiao , C. Shen , K. Wang , T. Wu , C. Liu , Y. Li , X. Yu , J. Liu , Q. Dai , K. Pajdzik , C. Ye , R. Ge , B. Gao , J. Yu , S. Sun , M. Chen , J. Chen , C. He , Nat. Cell Biol. 2023, 25, 1359. 37640841 10.1038/s41556-023-01213-w PMC10495261

[29] J. Chen , Environ. Toxicol. 2023, 38, 2545. 37471637 10.1002/tox.23883

[30] D. Dominissini , S. Moshitch‐Moshkovitz , S. Schwartz , M. Salmon‐Divon , L. Ungar , S. Osenberg , K. Cesarkas , J. Jacob‐Hirsch , N. Amariglio , M. Kupiec , R. Sorek , G. Rechavi , Nature 2012, 485, 201. 22575960 10.1038/nature11112

[31] M. Chen , L. Wei , C.‐T. Law , F. H.‐C. Tsang , J. Shen , C. L.‐H. Cheng , L.‐H. Tsang , D. W.‐H. Ho , D. K.‐C. Chiu , J. M.‐F. Lee , C. C.‐L. Wong , I. O.‐L. Ng , C.‐M. Wong , Hepatology 2018, 67, 2254. 29171881 10.1002/hep.29683

[32] M. Mei , L.u Chen , J. Godfrey , J. Song , C. Egelston , S. Puverel , L. E. Budde , S. Armenian , L. Nikolaenko , M. Nwangwu , W. Guo , L. Gao , P. Lee , R. Chen , S. Daniels , N. Kennedy , L. Peters , J. Zain , S. Rosen , S. Forman , L. Popplewell , L. Kwak , A. F. Herrera , Blood 2023, 142, 1359. 37339586 10.1182/blood.2023020485 PMC11561539

[33] Z. Chen , C. Peng , C. Jin , Y.e Wang , T. Wang , P. Yang , W. Peng , Q. Sun , H. Xu , H. Nie , X. Wang , J. Tang , Y. Sun , Y. Feng , Adv. Sci. 2025, 12, 2401964. 10.1002/advs.202401964 PMC11967759 39928532

[34] K. Gong , M. Wang , Q. Duan , G. Li , D. Yong , C. Ren , Y. Li , Q. Zhang , Z. Wang , T. Sun , H. Zhang , Q. Tu , C. Wu , J. Fu , A. Li , C. Song , Y. Zhang , R. Li , Metab. Eng. 2023, 75, 131. 36528227 10.1016/j.ymben.2022.12.002

[35] F. Wang , Y. Jin , M. Wang , H.‐Y. Luo , W.‐J. Fang , Y.‐N. Wang , Y.‐X. Chen , R.‐J. Huang , W.‐L. Guan , J.‐B. Li , Y.‐H. Li , F.‐H. Wang , X.‐H. Hu , Y.‐Q. Zhang , M.‐Z. Qiu , L.‐L. Liu , Z.‐X. Wang , C. Ren , D.‐S. Wang , D.‐S. Zhang , Z.‐Q. Wang , W.‐T. Liao , L. Tian , Q. Zhao , R.‐H. Xu , Nat. Med. 2024, 30, 1035. 38438735 10.1038/s41591-024-02813-1

[36] C. Hu , Z. Xin , X. Sun , Y. Hu , C. Zhang , R. Yan , Y. Wang , M. Lu , J. Huang , X. Du , B. Xing , X. Liu , Clin Cancer Res. 2023, 42, 108. 10.1186/s13046-023-02656-7 PMC10150531 37122003

[37] K. Li , K. Zhang , H. Wang , Y. Wu , N. Chen , J. Chen , C. Qiu , P. Cai , M. Li , X. Liang , D. Su , Metabolism 2021, 114, 154349. 32888949 10.1016/j.metabol.2020.154349

[38] Y. Wang , K. Su , C. Wang , T. Deng , X. Liu , S. Sun , Y. Jiang , C. Zhang , B. Xing , X. Du , Cell Death Dis. 2024, 15, 545. 39085201 10.1038/s41419-024-06951-9 PMC11291975

[39] W. Zhao , C. Ouyang , L. Zhang , J. Wang , J. Zhang , Y. Zhang , C. Huang , Q. Xiao , B. Jiang , F. Lin , C. Zhang , M. Zhu , C. Xie , X.i Huang , B. Zhang , W. Zhao , J. He , S. Chen , X. Liu , D. Lin , Q. Li , Z. Wang , Nat. Commun. 2024, 15, 7455. 39198451 10.1038/s41467-024-51444-0 PMC11358276

[40] Y. Yang , J. Cai , X. Yang , K. Wang , K. Sun , Z. Yang , L. Zhang , L.u Yang , C. Gu , X. Huang , Z. Wang , X. Zhu , Mol. Ther. 2022, 30, 2342. 35192934 10.1016/j.ymthe.2022.02.021 PMC9171149

[41] A. F. Thomas , G. L. Kelly , A. Strasser , Cell Death Differ. 2022, 29, 961. 35396345 10.1038/s41418-022-00996-z PMC9090748

[42] Q. Zhao , Y. Bi , J. Guo , Y. Liu , J. Zhong , Y. Liu , L. Pan , Y. Guo , Y. Tan , X. Yu , Phytomedicine 2021, 80, 153399. 33202325 10.1016/j.phymed.2020.153399

[43] F. A. Sinicrope , R. L. Rego , K. Okumura , N. R. Foster , M. J. O'Connell , D. J. Sargent , H. E. Windschitl , Clin. Cancer Res. 2008, 14, 5810. 18794091 10.1158/1078-0432.CCR-07-5202 PMC2948480

[44] S. Bharati , M. Lev , Chest 1986, 90, 861. 3780332 10.1378/chest.90.6.861

[45] A. Cano‐González , M. Mauro‐Lizcano , D. Iglesias‐Serret , J. Gil , A. López‐Rivas , Cell Death Dis. 2018, 9, 134. 29374147 10.1038/s41419-017-0164-7 PMC5833688

[46] S. Arai , A. Varkaris , M. Nouri , S. Chen , L. Xie , S. P. Balk , ELife 2020, 9, 54954. 10.7554/eLife.54954 PMC7297531 32484436

[47] E. Oda , R. Ohki , H. Murasawa , J. Nemoto , T. Shibue , T. Yamashita , T. Tokino , T. Taniguchi , N. Tanaka , Science 2000, 288, 1053. 10807576 10.1126/science.288.5468.1053

[48] Q. Xue , R. Kang , D. J. Klionsky , D. Tang , J. Liu , X. Chen , Autophagy 2023, 19, 2175. 37055935 10.1080/15548627.2023.2200554 PMC10351475

[49] A. Champhekar , R. Heymans , J. Saco , G. Turon Font , C. Gonzalez , A. Gao , J. Pham , J. Lee , R. Maryoung , E. Medina , K. M. Campbell , D. Karin , D. Austin , R. Damioseaux , A. Ribas , Molecular Cancer 2023, 22, 165. 37803324 10.1186/s12943-023-01868-x PMC10557262

[50] L. Shi , H. Yan , S. An , M. Shen , W. Jia , R. Zhang , L.i Zhao , G. Huang , J. Liu , Mol. Oncol. 2019, 13, 358. 30443978 10.1002/1878-0261.12408 PMC6360364

