# Supplementary material for: Deacetylation of ACLY Mediates RNA M6A‐Modification of NOXA and Promotes Chemoresistance of Colorectal Cancer
Source: Adv Sci (Weinh). 2025 Oct 2;12(47):e03323. doi: 10.1002/advs.202503323 (PMC12713065; doi:10.1002/advs.202503323)
Supplement: Supplementary file 1 — Supporting Information [file ADVS-12-e03323-s001.docx]

Supporting Information

**Deacetylation of ACLY Mediates RNA m^6^A-Modification of NOXA and Promotes Chemoresistance of** **Colorectal Cancer**

*Jun Wen, Mengqin Shen, Haitao Zhao, Liu Liu, Qian Hua, Xiaoping Zhao, Jianjun Liu*, Haizhong Feng^*^, Gang Huang^*^*

**Figure S1 Page 2**

**Figure S2 Page 4**

**Figure S3 Page 5**

**Figure S4 Page 6**

**Figure S5 Page 7**

**Figure S6 Page 9**

**Figure S7 Page 10**

**Table S1 Page 12**

**Table S2 Page 12**

**Table S3 Page 13**

**Table S4 Page 14**


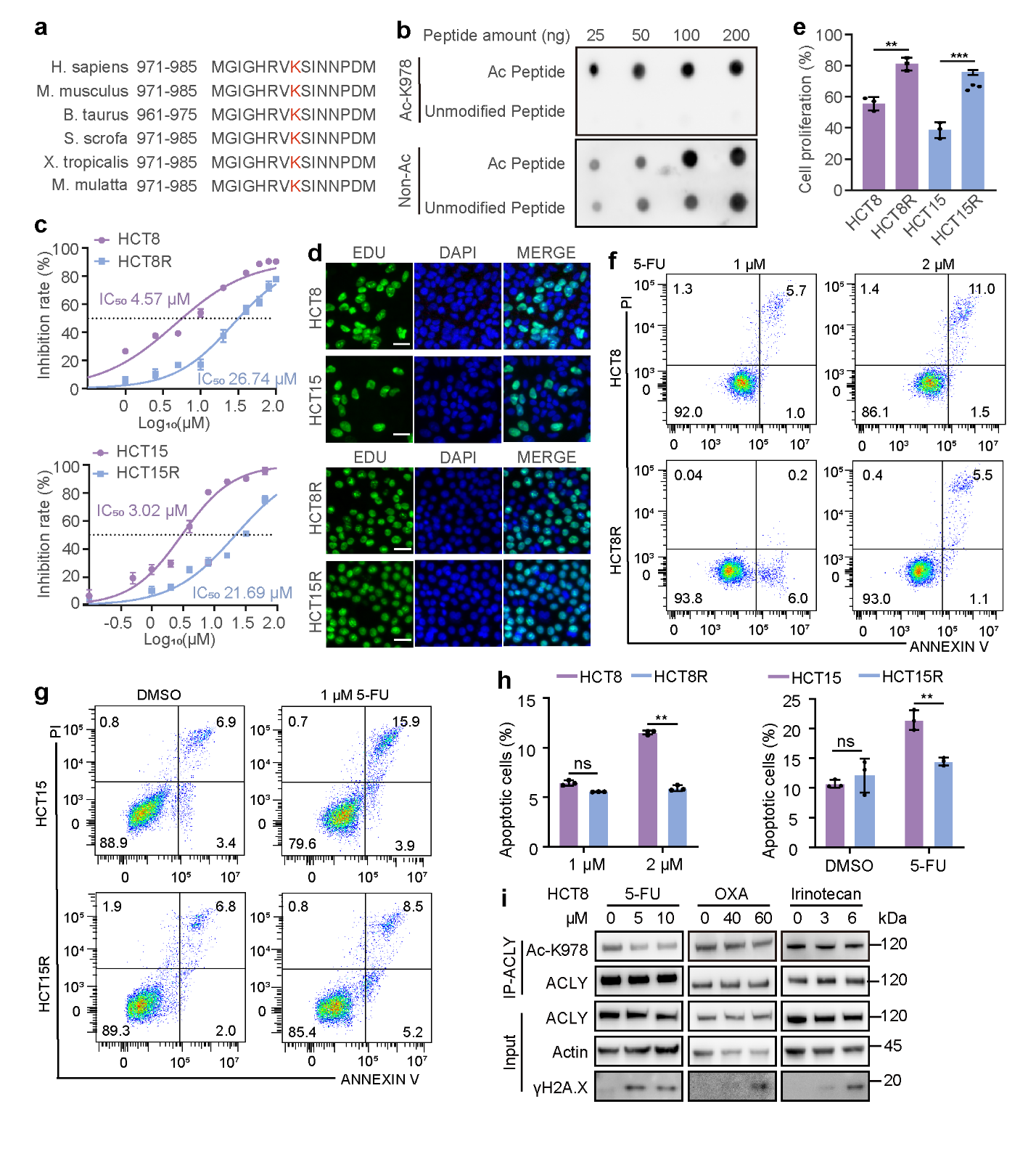


**Figure S1.** ACLY-K978 acetylation is downregulated in chemoresistant CRC. a) Sequences surrounding lysine (K) 978 in ACLY among six species were aligned. Lysine 978 was colored in red. b) Dot blot assay to identify the specificity of ACLY-K978ac antibody and ACLY-K978non-ac antibody. c) 5-FU dose-response curves on the parental and 5-FU resistant CRC cell lines over 48 hours (n = 3). d, e) Edu staining of colorectal cancer cells (parental and 5-FU resistant cells). Cells were treated with 2 μM 5-FU for 48 hours and then labeled with anti-EdU (green) antibodies, as well as Hoechst 33342 for nuclear staining (blue). Scale bar represents 25 µm. Representative images are shown in (e). f-h) Colorectal cancer parental cells and 5-FU resistant cells were treated with DMSO or 5-FU for 48 hours. Cells were subjected to Annexin V/PI staining (n = 3). i) Western blot analysis of K978 acetylation level in HCT8 cells treated with different concentrations of 5-FU, oxaliplatin, or irinotecan. Error bars indicate mean ± SD. **P* < 0.05, ***P* < 0.01, and ****P* < 0.001.


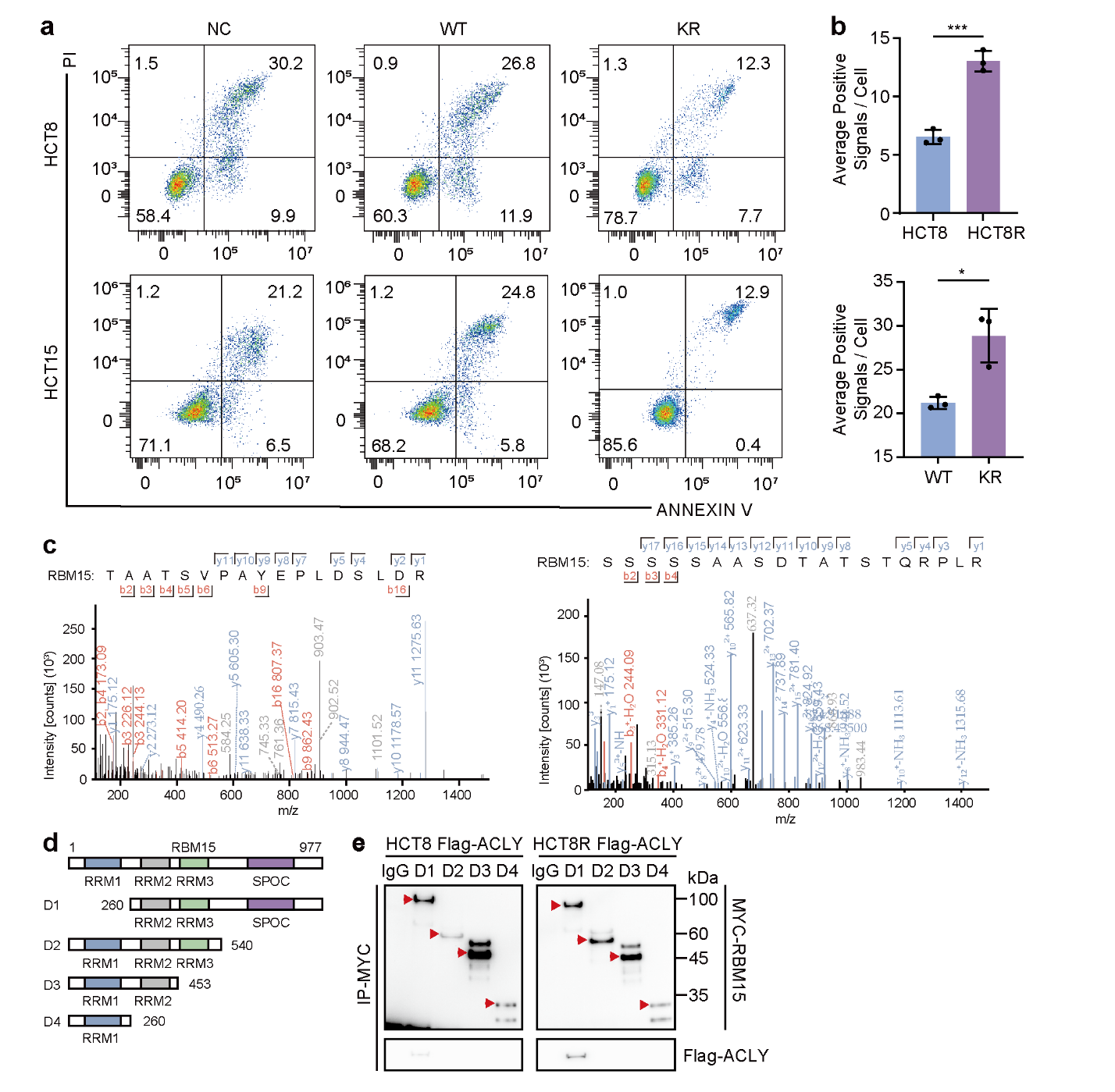


**Figure S2.** a) Apoptotic cells induced by 5-FU treatment were analyzed by flow cytometry (n = 3). b) Corresponding quantification of PLA signals (Figure 3h, i) are displayed. Error bars indicate mean ± SD. **P* < 0.05 and ****P* < 0.001. c) Mass spectrometry spectrum showing the peptide of RBM15 that binds to ACLY protein. d) Schematic of MYC-RBM15 (full length) and series of deletion mutants is shown. e) Immunoprecipitation and western blot assay were conducted using recombinant MYC-RBM15 deletion mutants (D1-D4) and Flag-ACLY overexpressed in HCT8 and HCT8R cells.


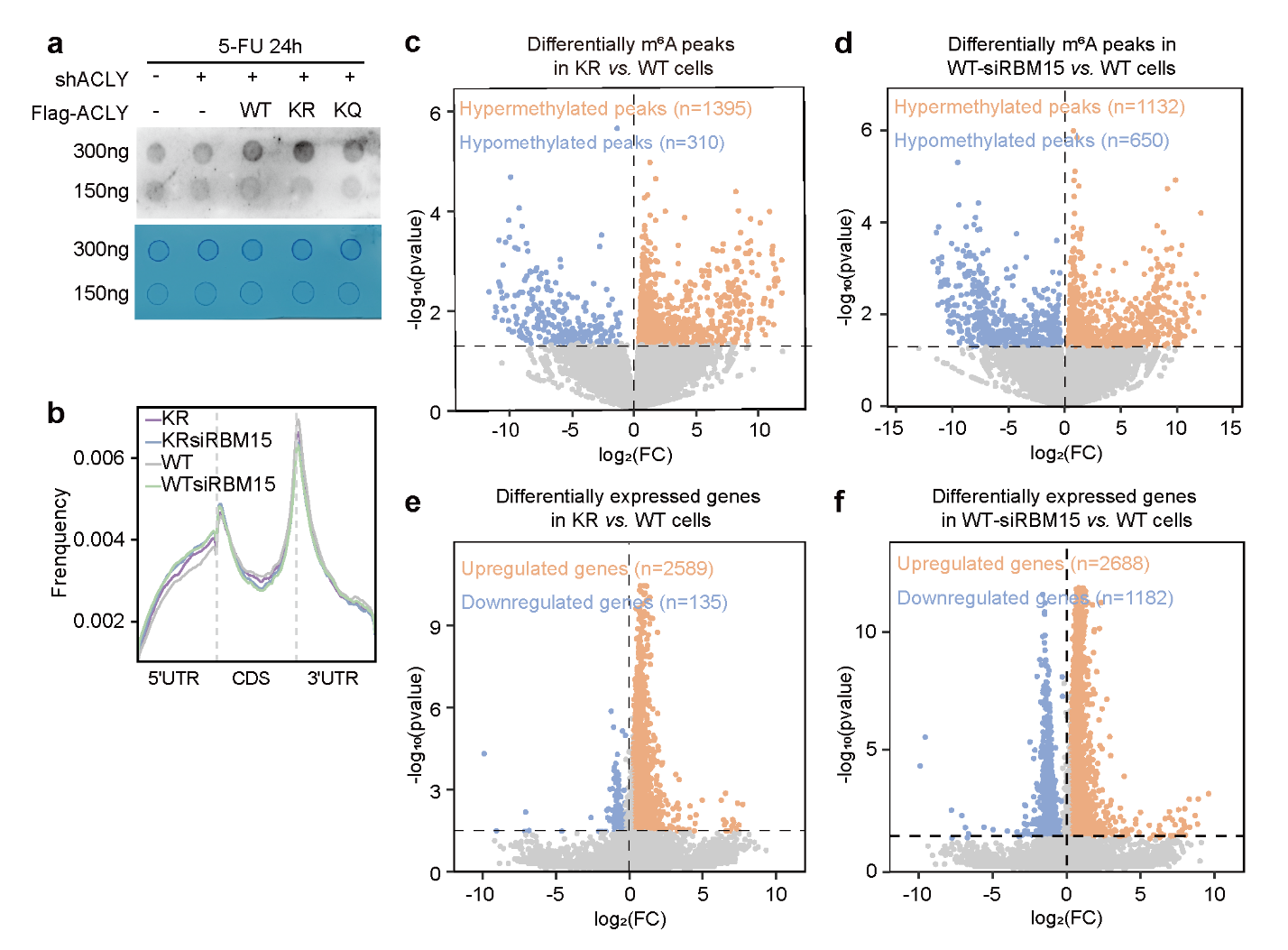


**Figure S3.** The analysis of meRIP sequence. a) Dot blot assay showing the verification of m^6^A abundance (upper: RNA dot blot, below: methylene blue). b) Overall m^6^A frequencies along indicated different regions of mRNAs in WT, KR, and relative RBM15 knockdown cells. c) Volcano plot of hyper- and hypomethylated peaks in KR versus WT cells. d) Volcano plot of hyper- and hypomethylated peaks in WT-siRBM15 versus WT cells. e) Volcano plot of up- and downregulated genes in KR versus WT cells. f) Volcano plot of up- and downregulated genes in WT-siRBM15 versus WT cells.

**
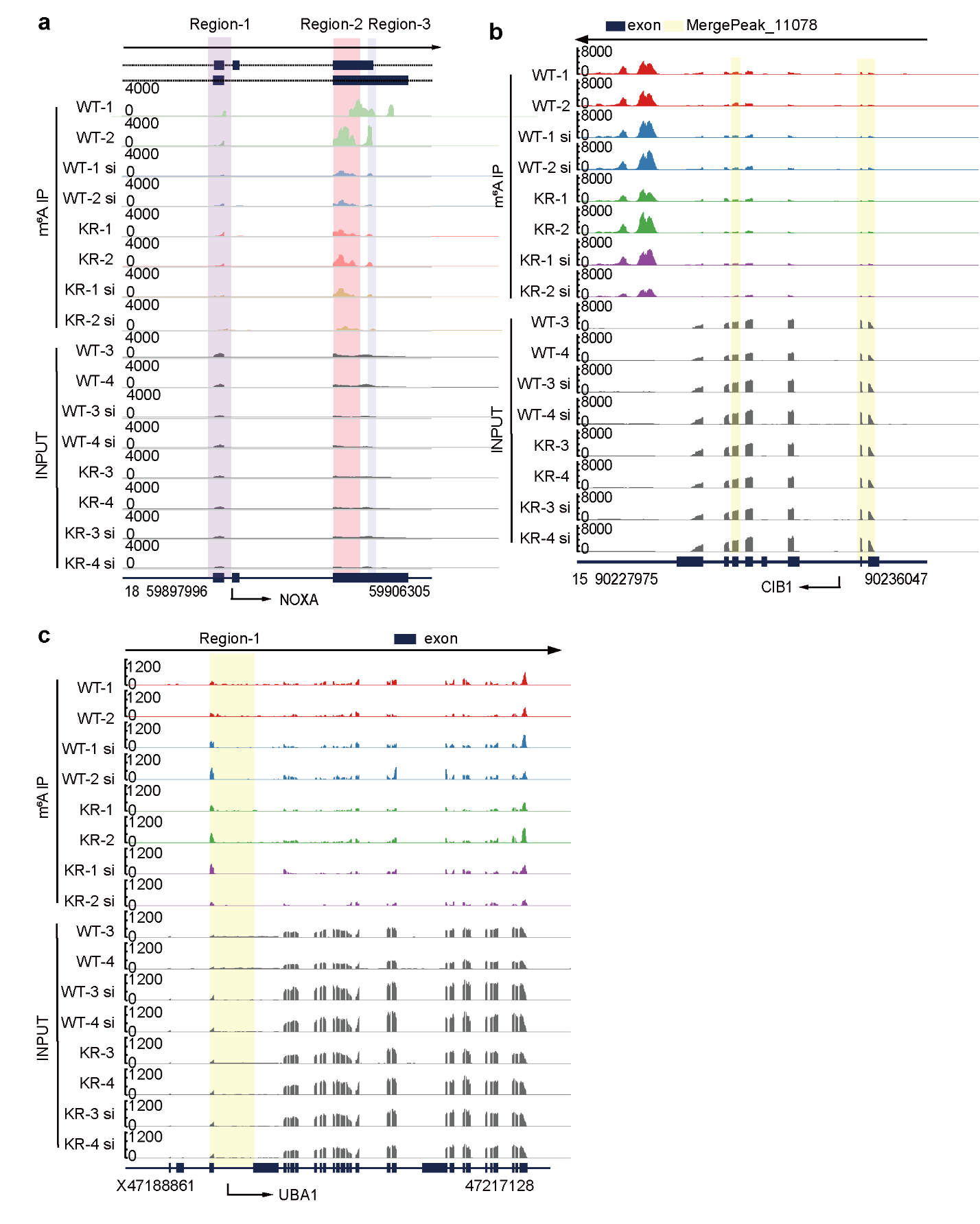
**

**Figure S4.** a-c) IVG screenshot showing input RNA sequence and m^6^A sequence signal profiles of NOXA (a), CIB1 (b) and UBA1 (c) gene loci in WT, KR, and relative RBM15 knockdown group (eg. named as WT-1 si, KR-1 si).

**
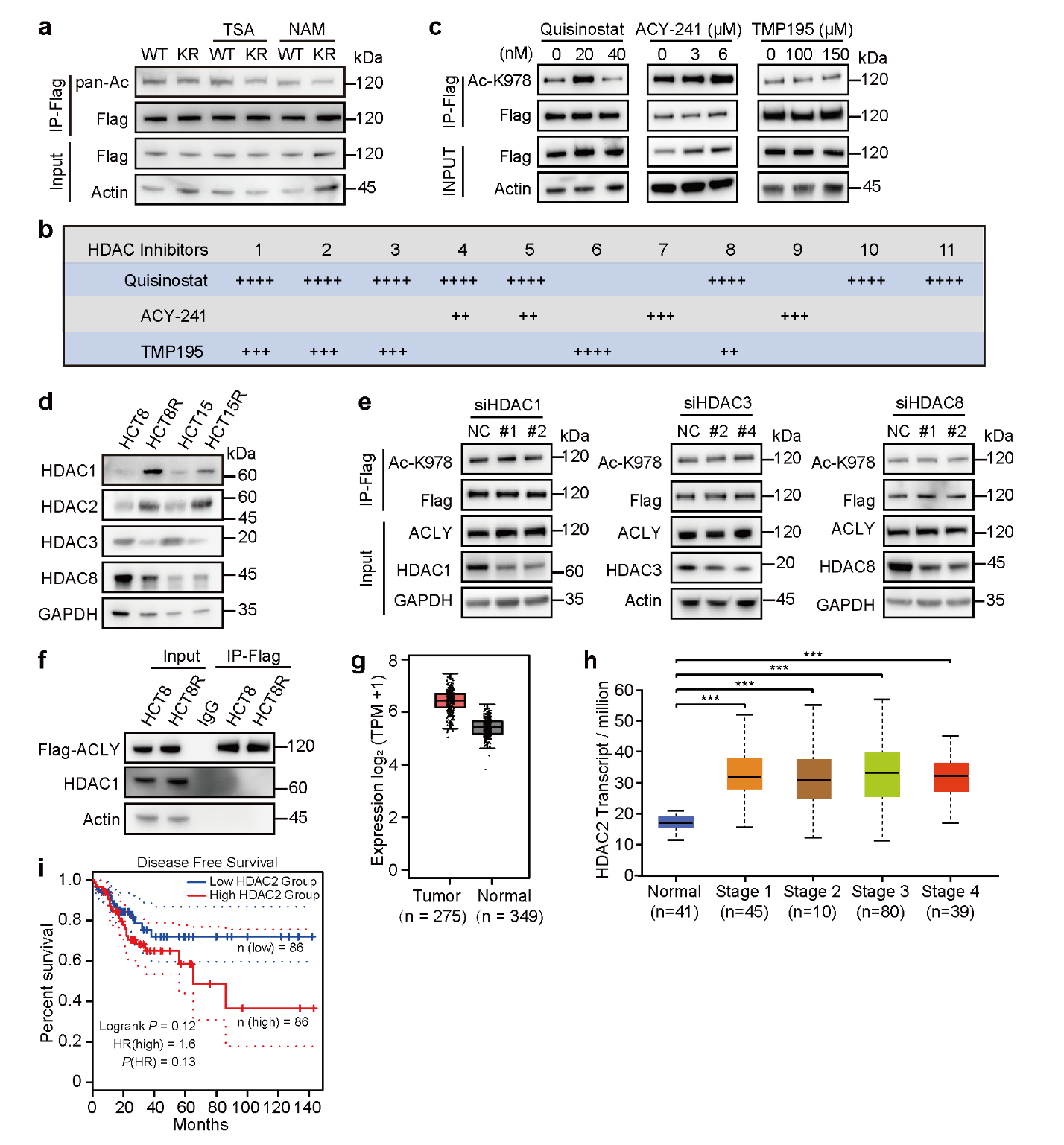
**

**Figure S5.** Screening the deacetylases of ACLY-K978. a) Western blot to detect the ACLY-K978 acetylation level after treatment with DMSO, NAM, or TSA. b) HDAC selective inhibitors and their targets. c) Western blot analysis of K978 acetylation level after HDAC selective inhibitors treatment. c) Western blot analysis of K978 acetylation level when HDAC1/3/8 knockdown. Each Western blot was independently repeated three times. d) Western blot analysis to detect the expression level of HDAC family proteins in CRC cell lines and 5-FU resistant CRC cell lines. e) Immunoprecipitation and western blot assay to detect interaction between ACLY and HDAC1 in HCT8 and HCTR cells. f) Immunoprecipitation and western blot assay to detect interaction between ACLY and HDAC1 in HCT8 and HCTR cells. g) Comparison of HDAC2 mRNA expression between normal tissue (n = 349) and CRC (n = 275) from GEPIA2 dataset. h) HDAC2 mRNA level in different tumor grades of CRC from UALCAN dataset. i) Kaplan-Meier disease free survival (DFS) analysis using the expression level of HDCA2 in 172 CRC patients with microsatellite stability (MSS) (from GEPIA2 dataset), log-rank test. Error bars indicate mean ± SD. For comparisons among multiple groups, one-way ANOVA was employed. ****P* < 0.001.


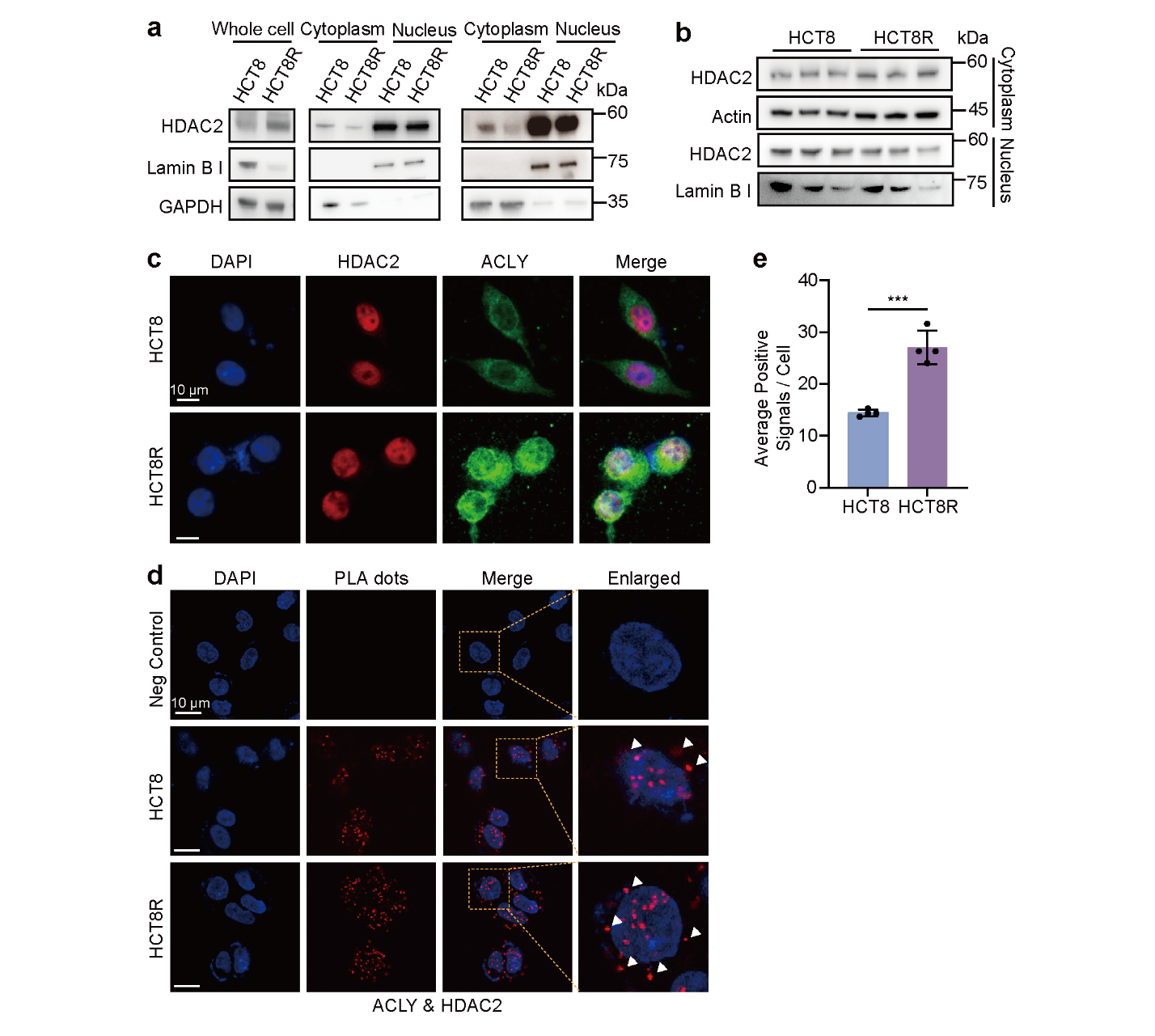


**Figure S6.** a) Western blot to detect HDAC2 protein localization in HCT8 and HCT8R cells. b, c) Western blot analysis to detect the expression levels of HDAC2 protein in the cytoplasmic (b) and nuclear (c) fractions, respectively. in HCT8 and HCT8R cells. d) Immunofluorescence staining showing the subcellular localization of HDAC2 (red) and ACLY (green) in HCT8 and 5-FU resistant HCT8R cells (scale bars, 10 μm). e) In situ proximity ligation assay (PLA) demonstrated interaction between ACLY and HDAC2 in HCT8 and HCT8R cells. Positive PLA signals (white arrows) showed ACLY/HDAC2 complex, shown as red clusters, with cell nuclei stained blue (scale bars, 10 μm). f) Corresponding quantification of PLA signals (e) is displayed (n = 4). Error bars represent the mean ± SD. ****P* < 0.001.


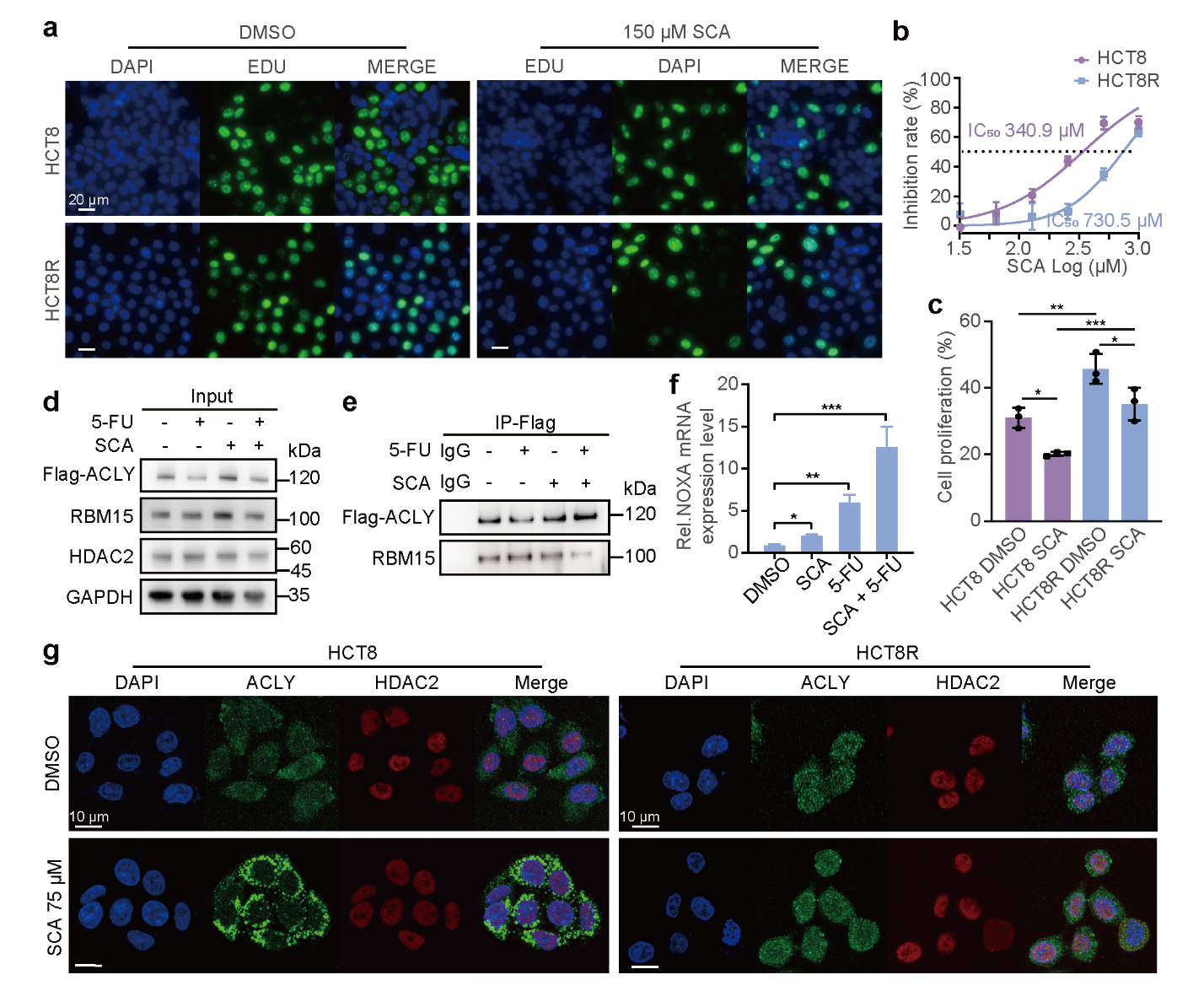


**Figure S7.** a) Edu staining of colorectal cancer cells (HCT8 and HCT8R cells). Cells were treated with DMSO or 150 μM HDAC2 inhibitor Santacruzamate A (SCA) for 48 hours and then labeled with anti-EdU (green) antibodies, as well as Hoechst 33342 for nuclear staining (blue). Scale bar represents 20 µm. b) SCA dose-response curves on HCT8 and HCT8R cells over 48 hours (n = 6). c) Corresponding quantification of cell proliferation rates (a) are displayed (n = 3). d, e) The immunoprecipitation (IP) assay to detect interaction between ACLY and RBM15 in HCT8/shACLY cells stably expressing ACLY after treatment with 5-FU or SCA for 48 hours. f) The expression level of NOXA mRNA measured in HCT8 cells treated with 5-FU or SCA (n =3 ). g) Immunofluorescence staining assays for ACLY (green), HDAC2 (red) and DAPI (blue) were conducted in HCT8 and HCT8R cells treated with SCA (scale bars, 10 μm). Error bars indicate mean ± SD. For comparisons among multiple groups, one-way ANOVA was employed. **P* < 0.05, ***P* < 0.01, and ****P* < 0.001.

**Table S1.** The global acetylation level ratio in sensitive and non-sensitive samples

| Sample Number | ACLY-K978 Acetylation Ratio (a) | ACLY Protein Ratio (b) | Normalized Ratio (a/b) |
| --- | --- | --- | --- |
| Patient 1/5 | 1.08 | 0.869 | 1.243 |
| Patient 2/6 | 1.21 | 0.852 | 1.420 |
| Patient 3/7 | 1.13 | 0.914 | 1.238 |
| Patient 4/8 | 0.99 | 0.831 | 1.191 |

**Table S2.** Detailed Patient Data for acetylation of K978 and clinicopathological parameters of colorectal cancer patients

| Characteristics | All cases | High | Low | *P* value |
| --- | --- | --- | --- | --- |
| Participants | 65 | 20 | 45 |  |
| Age (years) | | | | |
| <60 | 29 | 12 | 17 | 0.0914 |
| ≥60 | 36 | 8 | 28 |  |
| Gender |  |  |  |  |
| Male | 36 | 14 | 22 | 0.1093 |
| Female | 29 | 6 | 23 |  |
| TNM stages |  |  |  |  |
| 1-2 | 24 | 9 | 15 | 0.3650 |
| 3-4 | 41 | 11 | 30 |  |
| Tumor size (cm) |  |  |  |  |
| ≤5 | 41 | 12 | 29 | 0.7304 |
| >5 | 24 | 8 | 16 |  |
| Lymph node  metastasis |  |  |  |  |
| Negative | 36 | 10 | 26 | 0.5581 |
| Positive | 29 | 10 | 19 |  |

**Table S3.** Sequences of siRNAs, primers

| Usage | Target genes | Sequence (5’-3’) |
| --- | --- | --- |
| siRNA for candidate proteins knock down. | Negative Control | UUCUCCGAACGUGUCACGUTT |
|  | ACLY | AUCAAACGUCGUGGAAAA |
|  | RBM15 #1 | GCACCAUAGACUACCGAAATT |
|  | RBM15 #2 | GGACCUUUAUCCUGACUCUTT |
|  | HDAC1 #1 | ACUAUGGUCUCUACCGAAA |
|  | HDAC1 #2 | GCAAGUAUUAUGCUGUUAA |
|  | HDAC2 #1 | UCUAACAGUCAAAGGUCAUGCUAAA |
|  | HDAC2 #2 | GAAGAUCCAGACAAGAGAAUUUCUA |
|  | HDAC3 #2 | CACCCAAUGAGUUCUAUGATT |
|  | HDAC3 #4 | CAGCUGAACAACAAGAUCUTT |
|  | HDAC8 #1 | GCAGAUGAGGAUAGUUAAGTT |
|  | HDAC8 #2 | GGUGUACAUAGCCUUUAAUTT |
| qRT-PCR primers | Beta-Actin-F | CCTTCCTGGGCATGGAGTC |
|  | Beta-Actin-R | TGATCTTCATTGTGCTGGGTG |
|  | ACLY-F | ATCGGTTCAAGTATGCTCGGG |
|  | ACLY-R | GACCAAGTTTTCCACGACGTT |
|  | RBM15-F | ACGACCCGCAACAATGAAG |
|  | RBM15-R | GGAAGTCGAGTCCTCACCAC |
|  | CIB1-F | GGAGACCGATCGCTGAGAAC |
|  | CIB1-R | TAAGGCTGTCTTTGGCTGGG |
|  | NOXA-F | GGAGCTGGAAGTCGAGTGTG |
|  | NOXA-R | GAAACGTGCACCTCCTGAGA |
|  | UBA1-F | GAACCGGCATTGATGTCCAGC |
|  | UBA1-R | TGTCTGGAGCCGCTTCATTG |
| **Continued** **Table S3** | | |
| meRIP-qPCR primers | CIB1-1-F | ATACAGGCGAGTCCCACACC |
|  | CIB1-1-R | CGGGAAGTCAAGCCGCAC |
|  | CIB1-2-F | GGCTTCCAGCCCAAGTTCAA |
|  | CIB1-2-R | GCTAAATGGTTGCCTGCGTT |
|  | NOXA-1-F | GCTCTGTAGCTGAGTGGGCG |
|  | NOXA-1-R | CCCCAGCGGGTCGGTA |
|  | NOXA-2-F | GCTCAGGAACCTGACTGCAT |
|  | NOXA-2-R | GAAACGTGCACCTCCTGAGA |
|  | NOXA-3-F | ACTTGGGGCCAGTAAATCAGT |
|  | NOXA-3-R | CCCTGGTTATACAAAATCCCCA |
|  | UBA1-F | CAGGGACAACCACAACCACA |
|  | UBA1-R | CTCAAGGAGCCGAAGCCAA |

**Table S4.** Antibody used in this study

| Antibody | Company | Experiments |
| --- | --- | --- |
| Beta-Actin-HRP | Beyotime, China | WB 1:2500 |
| Lamine B I Mouse | Proteintech, 66095 | WB 1:50000 |
| GAPDH Mouse | Proteintech, 60004 | WB 1:50000 |
| Flag Mouse | Sigma, F1804 | WB 1:1000 PLA 1:1000 |
| ACLY Rabbit | Proteintech, 15421 | WB 1:2000 IF 1:25  RIP 5μg |
| ACLY Mouse | Proteintech, 67166 | PLA 1:200 |
| ACLY-K978ac Rabbit | Shanghai HuiOu, China | WB 1:500 IHC 1:50 |
| Acetylated-Lysine (Pan-Ac) Rabbit | CST, 9441 | WB 1:1000 |
| Cleaved PARP Rabbit | CST, 5625 | WB 1:1000 |
| Cleaved caspase-3 Rabbit | CST, 9664 | WB 1:1000 IHC 1:200 |
| **Continued** **Table S4** | | |
| Cleaved caspase-6 Rabbit | CST, 9761 | WB 1:1000 |
| Cleaved caspase-9 Rabbit | CST, 9505 | WB 1:1000 |
| H2AX (Ser139) Rabbit | CST, 2577 | WB 1:1000 IF 1:500 |
| RBM15 Rabbit | Proteintech, 10587 | WB 1:5000 PLA 1:2000 RIP 5μg |
| m^6^A Rabbit | Synaptic systems, 202-003 | WB 1:2000 Dot blot 1:2000 |
| HDAC1 Rabbit | CST, 34589 | WB 1:1000 |
| HDAC2 Rabbit | Proteintech, 12922 | WB 1:5000 IP 0.5-4.0μg  PLA 1:2000 |
| HDAC3 Rabbit | CST, 85057 | WB 1:1000 |
| HDAC8 Rabbit | Abcam, ab187139 | WB 1:10000 |
| Ki67 | Proteintech, 27309 | IHC 1:5000 |
